# SiRCle (Signature Regulatory Clustering) model integration reveals mechanisms of phenotype regulation in renal cancer

**DOI:** 10.1186/s13073-024-01415-3

**Published:** 2024-12-04

**Authors:** Ariane Mora, Christina Schmidt, Brad Balderson, Christian Frezza, Mikael Bodén

**Affiliations:** 1 School of Chemistry and Molecular Biosciences, University of Queensland, Molecular Biosciences Building 76, St Lucia, QLD 4072 Australia; 2 Medical Research Council Cancer Unit, Hutchison/MRC Research Centre, University of Cambridge, Cambridge Biomedical Campus, Box 197, Cambridge, CB2 0X2 UK; 3 University of Cologne, Faculty of Medicine and University Hospital Cologne, Institute for Metabolomics in Ageing, Cluster of Excellence Cellular Stress Responses in Aging-associated Diseases (CECAD), Joseph-Stelzmann-Str. 26, Cologne, 50931 Germany; 4 University of Cologne, Faculty of Mathematics and Natural Sciences, Institute of Genetics, Cluster of Excellence Cellular Stress Responses in Aging-associated Diseases (CECAD), Cologne, Germany

**Keywords:** Integration, Multi-omics, Regulation, Machine learning, Variational autoencoder, Clear cell renal cell carcinoma, PanCan, Metabolism

## Abstract

**Background:**

Clear cell renal cell carcinoma (ccRCC) tumours develop and progress via complex remodelling of the kidney epigenome, transcriptome, proteome and metabolome. Given the subsequent tumour and inter-patient heterogeneity, drug-based treatments report limited success, calling for multi-omics studies to extract regulatory relationships, and ultimately, to develop targeted therapies. Yet, methods for multi-omics integration to reveal mechanisms of phenotype regulation are lacking.

**Methods:**

Here, we present SiRCle (**Si**gnature **R**egulatory **Cl**ust**e**ring), a method to integrate DNA methylation, RNA-seq and proteomics data at the gene level by following central dogma of biology, i.e. genetic information proceeds from DNA, to RNA, to protein. To identify regulatory clusters across the different omics layers, we group genes based on the layer where the gene’s dysregulation first occurred. We combine the SiRCle clusters with a variational autoencoder (VAE) to reveal key features from omics’ data for each SiRCle cluster and compare patient subpopulations in a ccRCC and a PanCan cohort.

**Results:**

Applying SiRCle to a ccRCC cohort, we showed that glycolysis is upregulated by DNA hypomethylation, whilst mitochondrial enzymes and respiratory chain complexes are translationally suppressed. Additionally, we identify metabolic enzymes associated with survival along with the possible molecular driver behind the gene’s perturbations. By using the VAE to integrate omics’ data followed by statistical comparisons between tumour stages on the integrated space, we found a stage-dependent downregulation of proximal renal tubule genes, hinting at a loss of cellular identity in cancer cells. We also identified the regulatory layers responsible for their suppression. Lastly, we applied SiRCle to a PanCan cohort and found common signatures across ccRCC and PanCan in addition to the regulatory layer that defines tissue identity.

**Conclusions:**

Our results highlight SiRCle’s ability to reveal mechanisms of phenotype regulation in cancer, both specifically in ccRCC and broadly in a PanCan context. SiRCle ranks genes according to biological features. https://github.com/ArianeMora/SiRCle_multiomics_integration.

**Supplementary Information:**

The online version contains supplementary material available at 10.1186/s13073-024-01415-3.

## Background

Clear cell renal cell carcinoma (ccRCC) is the most prevalent form of kidney cancer and accounts for 70% of renal malignancies [1]. It is now established that most ccRCC cases are driven by the loss of the Von Hippel-Lindau (VHL) tumour suppressor gene, in turn leading to the activation of the transcription factor (TF) hypoxia-inducible factors (HIFs) [2]. Although the activation of the HIF pathway is considered to be a driving event in VHL mutant ccRCCs, VHL mutations alone are insufficient for ccRCC formation and cooperating mutations such as PBRM1, BAP1, KDM5C and SETD2 are necessary for full transformation [3]. Hence, ccRCC transformation and progression have been reported to affect multiple regulatory layers, beyond transcription, including the epigenome, via changes in DNA methylation of CpG islands [4], the proteome, via alterations in the mammalian target of rapamycin (mTOR) pathway [3] that in turn affects protein synthesis [5], and the metabolome, whose dysregulation is considered a hallmark of ccRCC [6–8].

Due to the complexity of gene regulation in ccRCC, molecular analyses for patient stratification and drug treatment based exclusively on genomic or transcriptomics analysis have been unsuccessful [9]. For example, protein expression via (post-) translational regulation is altered in cancer [10] and there is a lack of correlation between transcriptome and proteome [11]. Hence, it is difficult to predict pathway activity such as TFs driving disease states from transcriptomics data alone, suggesting that data from distinct regulatory layers need to be analysed jointly. Furthermore, patients’ responses to different treatments can be dependent on the mutational and methylation landscape of the patients [12]. To overcome these issues, consortia such as CPTAC (Clinical Proteomic Tumor Analysis Consortium) are leading efforts to produce multi-omics datasets with various patient demographics [13]. Clark et al. reported the first extensive dataset of ccRCC patients that includes tumour and normal RNA-seq and proteomics data, and tumour DNA methylation data [14]. Clark et al. proposed a stratification of ccRCC patients for personalised therapeutic interventions based on the differential RNA and protein profiles of the patients, along with the copy number variation and phosphoproteome [14]. However, it remains unclear if in ccRCC a gene of interest is regulated at the level of DNA-methylation, transcription or translation, and changes at what regulatory level (henceforth “layer”) dictates the cellular phenotype of ccRCC. Addressing this question is vital to identify determinants that explain disease states and to subsequently develop effective anticancer therapies.

Data integration is a core component of multi-omic analyses but remains a challenge owing to dataset heterogeneity, noise and non-linearities between genetic and epigenetic interactions [15]. Many approaches exist to integrate bulk multi-omic assays to understand cancer genomics; they are typically based on standard statistical methods, clustering or linear matrix factorisations [16, 17]. Whilst approaches exist to integrate data across a broad range of modalities, such as MixOmics [18], others are designed specifically to integrate assays such as copy number variation, proteomics and gene expression [16, 17], which has the benefit of imposing knowledge of the flow of biological information into the methods design. An alternative approach to including biological knowledge is by incorporating network structure into the analyses [19–21]. Recently, deep learning methods, such as variational autoencoders (VAEs) [22], have been applied to detect non-linear patterns across patients in single genomic data types in cancer patients [23, 24]. VAEs have been used for differential analysis, cancer subtype identification, data integration [25, 26], and survival analysis [26], using both bulk [23] and single cell [27] data, and on a variety of assays (ATAC [25], mRNA [23, 24, 28], DNA methylation [20]). Moreover, VAE applied to TCGA pan-cancer RNA-seq data enabled the separation of the data according to the underlying cancer type [23].

To date, studies involving GWAS (genome-wide association studies) data suggest statistics derived from principal component analysis (PCA) embeddings enable complex relationships to be identified, opening an avenue for multi-omic patient changes to be extracted [29, 30]. VAEs present a machine learning approach to extract latent features that summarise variation across the dataset [31], akin to PCA but with the capacity to capture non-linearities. Recent research suggests that latent embeddings can be used to identify co-regulated gene groups [32]; however, such analyses have yet to be adapted for application on cancer or patient data. Other integration methods enable biological signals to be extracted along latent embeddings, typically in the forms of correlative or linear relationships [33–38], statistics on these bulk-cancer embeddings are not often performed. Yet, new methods that can detect multi-omic changes between patient cohorts and subtypes are becoming increasingly important, with research indicating that patients’ demographics such as age [39–41], gender [42, 43] and ethnicity [44] impact tumour gene expression profile and stage [45], and mutational profile [46] affect patient survival.

Here, we present “**Si**gnature **R**egulatory **Cl**ust**e**ring” (SiRCle), a method for integrating DNA methylation, mRNA and protein data at the gene level to deconvolute dysregulation within and across possible regulatory layers (DNA methylation, transcription and/or translation). Using SiRCle, we disentangled the regulatory layer behind metabolic rewiring in ccRCC and showed that glycolysis is regulated by DNA hypomethylation, whilst mitochondrial enzymes and respiratory chain complexes are translationally suppressed. Moreover, we identified that HIF1A is likely driving expression changes in glycolytic enzymes. We also explored the heterogeneity between ccRCC patient subpopulations to identify cell-type specific markers associated with patients’ survival along with the possible regulatory layer behind the differences in gene expression. We found downregulation of proximal renal tubule genes hinting towards a loss of cellular identity in cancer cells in specific SiRCle clusters and hence identify the regulatory layers responsible. Together this demonstrates how we can use SiRCle to uncover drivers across regulatory layers that may explain distinct cohorts of ccRCC. Next, we applied SiRCle to a PanCan cohort and find common signatures across ccRCC and PanCan in addition to the regulatory layer that defines tissue identity. Our results highlight SiRCle’s ability to reveal potential mechanisms of phenotype regulation in cancer, both specifically in ccRCC and broadly in a PanCan (Pan Cancer) context. SiRCle is available as a Python and R package at https://github.com/ArianeMora/SiRCle_multiomics_integration.

## Methods

### PanCan data selection

Data were downloaded on July 21, 2023, from CPATC (Clinical Proteomic Tumor Analysis Consortium) portal from the CPTAC3 cohort with at least five cases and included a protein assembly. For these studies, the clinical and biospecimen data were downloaded along with the protein summary file, containing the processed and normalised protein data via the CPTAC data processing pipeline. The accompanying gene expression and DNA methylation were downloaded from TCGA (The Cancer Genome Atlas) by selecting CPTAC3 data, filtering for solid tissue normal samples, or primary tumour samples, transcriptome profiling counts data, and DNA methylation array. The data were downloaded 18th of July 2023. For the CPTAC3 ccRCC-ITH cohort [47], data were downloaded on August 11, 2024. The cancers were filtered to include only the following primary diseases: Acute Myeloid Leukaemia, Breast Invasive Carcinoma, Clear Cell Renal Cell Carcinoma, Head and Neck Squamous Cell Carcinoma, Lung Adenocarcinoma, Lung Squamous Cell Carcinoma, Pancreatic Ductal Adenocarcinoma and Uterine Corpus Endometrial Carcinoma omitting samples not associated with a primary disease or un-descriptive classifications such as “Not Clear Cell Renal Cell Carcinoma”. Of the selected cancers, only Clear Cell Renal Cell Carcinoma (PDC000127 [14] (Proteomic Data Commons)), Head and Neck Squamous Cell Carcinoma (PDC000221 [48]), Lung Adenocarcinoma (PDC000153 [49]), Lung Squamous Cell Carcinoma (PDC000234 [50]) and Pancreatic Ductal Adenocarcinoma (PDC000270 [51]) had RNAseq and DNA methylation data for both tumour and normal with sufficient sample size. Cases were retained for a cancer if the case had an entry in the clinical information supplied by the CPTAC and TCGA portals. The stages of the tumours were consolidated to four tumour stage classifications: (1) “TumorStage”, stage I (stage I, stage IA, stage IB, stage IA3), stage II (stage II, stage IIA, stage IIB), stage III (stage III, stage IIIA, stage IIIB) and stage IV (stage IV, stage IVA, stage IVB). This classification was further grouped into early (stage I and stage II) and late-stage (stage III and stage IV). We found many cases had multiple files associated; these were further filtered by the biospecimen type, reducing to only include solid tissue specimens. The final processed sample sizes after removing samples without at least one sample in DNA methylation, gene expression and protein data were Head and Neck Squamous Cell Carcinoma (*N* = 55), Lung Adenocarcinoma (*N* = 107), Lung Squamous Cell Carcinoma (*N* = 97), Pancreatic Ductal Adenocarcinoma (*N* = 58) and Clear Cell Renal Cell Carcinoma (*N* = 102). The original studies and collection of these samples can be found at https://www.cell.com/consortium/cptac and the processed data in addition to filtering scripts can be downloaded at https://github.com/ArianeMora/public_cancer_data.

### DNA methylation processing

Data were further filtered to check for outliers prior to running differential analyses. For the DNA methylation data, beta values of 1.0 were replaced with 0.999 and beta values of 0 were replaced with 0.001. CpGs with an average methylation across all samples of > 5% and < 95% were retained. Correlation between samples was then calculated using Pearson’s correlation and samples with a Pearson’s correlation > 3 s.d. from the median correlation for each sample type were removed (with the exception from Pancreatic Ductal Adenocarcinoma and Lung Adenocarcinoma where a cutoff of 2 s.d. was used as PCA showed outlier samples affected the PCs). CpG samples with missing data in 50% of samples were also removed, before null values were replaced with 0.001.

### Gene expression processing

For RNAseq data, genes with mean counts ≤ 10 across samples were removed before calculating the correlation between samples for each sample type (i.e. tumour and solid tissue normal); those with a median sample Pearson’s correlation > 3 s.d. from the median were removed. RNA samples with missing data in 50% of samples were also removed, before null values were replaced with 0 s. Each cancer was visually inspected using PCA to confirm separability within the cancer between tumour and normal samples. Finally, for patients where more than one sample passed the QC thresholds, only one sample was retained for tumour, normal, and DNA methylation and gene expression.

### Protein data processing

For the protein data, the data as processed by CPTAC were used; these are normalised and have been assigned to genes in a consistent fashion across cancers. For Lung Adenocarcinoma, samples with case IDs not fitting the standard convention were omitted namely those (i.e. 11LU013_Tumor_Protein_CPT0053040004, 11LU016_Tumor_Protein_CPT0052940004, 11LU022_Tumor_Protein_CPT0052170004, 11LU035_Tumor_Protein_CPT0051690004). Genes with 0 s in more than 50% of samples omitted. Gene wise correlation between samples for each sample type (i.e. tumour and solid tissue normal) was calculated using Pearson’s correlation. Samples with a median correlation less than the median minus 3 s.d. were removed. Missing protein data were imputed using DreamAI ensemble method [52] (https://github.com/WangLab-MSSM/DreamAI). Samples exhibited a high correlation post imputation, with tumour and normal samples clustering distinctly, and as such no protein samples were removed. The supplied protein names were mapped to hgnc symbols using biomart mappings (scibiomart, 1.0.2, https://github.com/ArianeMora/scibiomart), and for those without direct mappings were mapped using the external_synonym.

### PanCan (*Pan Cancer*) dataset generation

The filtered and imputed protein, gene expression and DNA methylation datasets were joined by gene name, ensembl ID and CpG ID respectively. An inner join was used to join on the ID for all datasets. The protein data was mean shifted to centre at 0 for each cancer, then when joined shifted by the minimum across all cancer datasets.

### PanCan differential analysis

Differential analysis was performed for all cancers. For the pan-can (Head and Neck Squamous Cell Carcinoma, Lung Adenocarcinoma, Lung Squamous Cell Carcinoma, Pancreatic Ductal Adenocarcinoma), disease was used as a factor in the differential analysis. For PanCan RNA-seq, DESeq2 [53] was used to calculate significant genes between tumour and normal samples using the design matrix included disease and condition, where condition indicates whether the sample was primary tumour or normal. For differential methylation analysis, we tested for differential methylation using lmFit and eBayes functions from limma [54] using *M* values as input (calculated as log_2_(beta/beta + 1)). Again, for the design matrix we used the disease as a factor in the pan-can analysis. Finally, for the proteomics differential expression analysis, we also use the limma pipeline using the same design matrix [52].

### ccRCC-ITH cohort dataset generation

All data for the ITH (intra-tumour heterogeneity) cohort [47] were downloaded from the CPTAC3 portal. Cases that were included in the original ccRCC cohort [14] were removed from the RNAseq and protein datasets, however, given there was only one normal DNA methylation sample profiled in the new cohort, we retained normal samples (12) that overlapped with the original ccRCC cohort [14], this left 229 of the 373 RNAseq samples, one of which was dropped due to low correlation with the other samples (mean rho = 0.17, C3L.00976_Tumor_RNA). For the protein dataset, there were originally 393 samples, which, once removed for the cases in the original dataset, was reduced to 240 samples, 145 of tumour and 95 of adjacent tissue. These were imputed and normalised as per the above pipeline. For DNA methylation, 222 tumour samples were assayed, which once filtered for the original cases, left 146 tumour DNA methylation samples. Two of these were removed as they had low correlation compared to the other samples (C3L.01951_Tumor_CpG, C3N.01989_Tumor_CpG); processing was performed as in the DNA methylation case for the original ccRCC cohort. Differential analysis was performed with the same pipeline as the ccRCC cohort for each data type.

### SiRCle (Signature Regulatory Clustering)

SiRCle input data are the same on each data layer, being a gene name and the results of a differential analysis, namely statistics and fold change/methylation difference. SiRCle provides maximal flexibility and requires the user to choose the “background method (BG)”, the thresholds/cutoffs of the input data (DNA-methylation, RNAseq and proteomics) and the “Regulation Grouping (RG)”.

The BG setting (Table 1) defines which genes are considered for the SiRCle clusters. For example, P&M&R is the most restrictive setting as here only genes detected in DNA-methylation, RNAseq and proteomics data layers are considered. Hence, the chosen BG method defines the number of genes included in the SiRCle input. Given that the proteomics data has the lowest coverage of the three input data layers, it has the biggest impact on the number of genes included in the SiRCle input.

**Table 1:** Background (BG) settings order from most restrictive (top) to least restrictive (bottom)

Parameter	Parameter meaning
P&M&R	Genes detected in Proteomics AND RNAseq AND DNA-Methylation
P&R	Genes detected in Proteomics AND RNAseq. Gene does not need to be detected in DNA-Methylation
P|(M&R)	Focus is on protein expression. Genes detected in Proteomics OR DNA-Methylation AND RNAseq
(P&M)|(P&R)	Gene is detected in Proteomics and one of the other two data layers: Proteomics AND DNA-Methylation OR Proteomics AND RNAseq
(P&M)|(P&R)|(M&R)	Genes detected in at least two of the three data layers: Proteomics AND DNA-Methylation OR Proteomics AND RNAseq OR DNA-Methylation AND RNAseq
P|R	Genes detected in on one of the two data layers: Proteomics OR RNAseq
P|M|R	Genes detected in one of the three data layers: Proteomics OR RNAseq OR DNA-Methylation

The user can set two different input thresholds, one for the differential expression, which includes “log_2_*FC*” for RNAseq and proteomics data layer and “Differential Methylation” for DNA-methylation, and one for “significance” (e.g. *p*.adj). The thresholds define what initial cluster the genes are placed in for each data layer (Table 2, columns 1–4), with “UP”/ “DOWN” meaning a gene is significantly up-/downregulated in the underlying comparison, “Hypermethylated”/ “Hypomethylated” meaning a gene’s methylation is significantly in-/decreased in the underlying comparison, and “No Change” includes genes that do not meet the significance and/or the fold change threshold, or dependent on the background (BG) setting were not detected (Table 2, columns 1–3). For the protein data layer, “No Change” is subdivided into “undetected”, which includes genes detected on other data layer, but not on the proteomics data layer, “not significant”, which includes proteins that do not meet the significance threshold set, “significant negative”, which includes proteins that do not meet the negative fold change threshold but meet the significance threshold, and “significant positive”, which includes proteins that do not meet the positive fold change threshold but meet the significance threshold (Table 2, column 4).

**Table 2:** Regulatory labels from the different grouping methods

Methylation	RNA-seq	Proteomics	Proteomics detection	RG2 Changes	RG3 Protein	RG4 Detection
Hypermethylation	DOWN	DOWN	DOWN	MDS	MDS	MDS
Hypermethylation	No Change	DOWN	DOWN	TMDS	TMDS	TMDS
Hypermethylation	UP	DOWN	DOWN	TPDE + TMDS	TMDS	TPDE + TMDS
Hypermethylation	DOWN	No Change	Not detected	MDS + TMDE	MDS	MDS
Hypermethylation	No Change	No Change	Not detected	None	None	None
Hypermethylation	UP	No Change	Not detected	TPDE + TMDS	TPDE	TPDE
Hypermethylation	DOWN	No Change	Not significant	MDS + TMDE	None	MDS + TMDE
Hypermethylation	No Change	No Change	Not significant	None	None	None
Hypermethylation	UP	No Change	Not significant	TPDE + TMDS	None	TPDE + TMDS
Hypermethylation	DOWN	No Change	Significant negative	MDS + TMDE	MDS	MDS
Hypermethylation	No Change	No Change	Significant negative	None	None	None
Hypermethylation	UP	No Change	Significant negative	TPDE + TMDS	TMDS	TPDE + TMDS
Hypermethylation	DOWN	No Change	Significant positive	MDS + TMDE	TMDE	MDS + TMDE
Hypermethylation	No Change	No Change	Significant positive	None	None	None
Hypermethylation	UP	No Change	Significant positive	TPDE + TMDS	TPDE	TPDE
Hypermethylation	DOWN	UP	UP	MDS + TMDE	TMDE	MDS + TMDE
Hypermethylation	No Change	UP	UP	TMDE	TMDE	TMDE
Hypermethylation	UP	UP	UP	TPDE	TPDE	TPDE
Hypomethylation	DOWN	DOWN	DOWN	TPDS	TPDS	TPDS
Hypomethylation	No Change	DOWN	DOWN	TMDS	TMDS	TMDS
Hypomethylation	UP	DOWN	DOWN	MDE + TMDS	TMDS	MDE + TMDS
Hypomethylation	DOWN	No Change	Not detected	TPDS + TMDE	TPDS	TPDS
Hypomethylation	No Change	No Change	Not detected	None	None	None
Hypomethylation	UP	No Change	Not detected	MDE + TMDS	MDE	MDE
Hypomethylation	DOWN	No Change	Not significant	TPDS + TMDE	None	TPDS + TMDE
Hypomethylation	No Change	No Change	Not significant	None	None	None
Hypomethylation	UP	No Change	Not significant	MDE + TMDS	None	MDE + TMDS
Hypomethylation	DOWN	No Change	Significant negative	TPDS + TMDE	TPDS	TPDS
Hypomethylation	No Change	No Change	Significant negative	None	None	None
Hypomethylation	UP	No Change	Significant negative	MDE + TMDS	TMDS	MDE + TMDS
Hypomethylation	DOWN	No Change	Significant positive	TPDS + TMDE	TMDE	TPDS + TMDE
Hypomethylation	No Change	No Change	Significant positive	None	None	None
Hypomethylation	UP	No Change	Significant positive	MDE + TMDS	MDE	MDE
Hypomethylation	DOWN	UP	UP	TPDS + TMDE	TMDE	TPDS + TMDE
Hypomethylation	No Change	UP	UP	TMDE	TMDE	TMDE
Hypomethylation	UP	UP	UP	MDE	MDE	MDE
No Change	DOWN	DOWN	DOWN	TPDS	TPDS	TPDS
No Change	No Change	DOWN	DOWN	TMDS	TMDS	TMDS
No Change	UP	DOWN	DOWN	TPDE + TMDS	TMDS	TPDE + TMDS
No Change	No Change	No Change	No Change	None	None	None
No Change	DOWN	No Change	Not detected	TPDS + TMDE	TPDS	TPDS
No Change	No Change	No Change	Not detected	None	None	None
No Change	UP	No Change	Not detected	TPDE + TMDS	TPDE	TPDE
No Change	DOWN	No Change	Not significant	TPDS + TMDE	None	TPDS + TMDE
No Change	No Change	No Change	Not significant	TPDS + TMDE	None	None
No Change	UP	No Change	Not significant	TPDE + TMDS	None	TPDE + TMDS
No Change	DOWN	No Change	Significant negative	TPDS + TMDE	TPDS	TPDS
No Change	UP	No Change	Significant negative	TPDE + TMDS	TMDS	TPDE + TMDS
No Change	DOWN	No Change	Significant positive	TPDS + TMDE	TMDE	TPDS + TMDE
No Change	UP	No Change	Significant positive	TPDE + TMDS	TPDE	TPDE
No Change	DOWN	UP	UP	TPDS + TMDE	TMDE	TPDS + TMDE
No Change	No Change	UP	UP	TMDE	TMDE	TMDE
No Change	UP	UP	UP	TPDE	TPDE	TPDE

The regulatory rules that we use to define the SiRCle flows are summarised into a smaller number of SiRCle clusters to facilitate downstream analysis and interpretation (Table 2, columns 5–7). Here, each SiRCle cluster reflects the regulation that ultimately results in changes to protein expression. Each change of regulation between the data layers defines the name of a SiRCle cluster. For example, “RG2 Changes” summarises the 54 possible flows into 10 SiRCle clusters considering any changes between the data layers, which means if there is a change of regulation between DNA-methylation and mRNA expression (e.g. Hypermethylation-DOWN) as well as a regulation between mRNA and protein expression (e.g. RNA = DOWN and Protein = UP) both of these regulations are reflected in the SiRCle clusters name Methylation-Driven-Suppression and Translation and post-transcriptional Modification Driven Enhancement (MDS + TMDE). “RG3 Protein” summarises the flows to only those that consider changes between mRNA expression and protein expression, meaning that in the example described, the gene falls into the cluster TMDE based on the secondary regulation at the translational level. Hence, “RG3 Protein” has 6 instead of 10 SiRCle clusters, which leads to larger clusters. Lastly, the RG “RG4 Detection” requires the gene to be detected on the protein layer to allow the changes between mRNA and protein layer (e.g. from “UP” on the mRNA to “No Change”/ “Not detected” on the protein layer) to be taken into account. If the BG method chosen enforces the protein to be detected (e.g. M&R&P), column “RG4 Detection” will include the same as “RG2 Changes” as the flow through “Not detected” cannot occur.

After performing the CpG analysis to identify differentially methylated CpGs using limma, CpGs were filtered to map only one CpG to a given gene, since SiRCle requires a single DNA methylation value to be assigned to a gene. This was done by grouping the CpG data by the gene they are annotated to by Illumina (https://support.illumina.com/array/array_kits/infiniummethylationepic-beadchip-kit/downloads.html). In detail, if there were less than three significant ($P<0.05$) CpGs, then the CpG with the greatest absolute change was selected. However, given some genes have many CpGs annotated to them, we opted to filter to only include genes where the direction of change was consistent across the changing CpGs. Specifically, if there were more than 3 CpGs with $p<0.5$, we tested if the majority (> 60%) of CpGs agreed in the “direction of change” (positive or negative) and if not all CpGs were omitted, otherwise, the CpG with the highest methylation difference was chosen. We chose a conservative approach to annotating the CpG changes to a gene as SiRCle aims to annotate the functional effect of a change in DNA methylation, as such we only include genes where the DNA methylation change is consistent (e.g. >60% agree). This choice is up to the user as the pre-filtering is a step before SiRCle. The choice of filter will depend strongly on the type of DNA methylation assay that has been performed.

Next, we performed the SiRCle clustering on the filtered CpG data, DE results from DEseq2 (RNA-seq Data) and the output from limma’s abundance analysis (proteomics data). The datasets were merged using the background method: P|(M&R) meaning a gene was retained if it was changed significantly ($p<0.05$) in at least two of the three input datasets (RNA-seq, proteomics, DNA methylation). Next, regulatory clusters were assigned using the cutoffs of adjusted *p* values < 0.05, and mRNA |${\text{log}}_{2}FC|>1.0$, protein ${|\text{log}}_{2}FC|>0.5$ and |DNA methylation beta difference|> 0.1. The choice of the cutoffs for DNA methylation, gene expression and protein changes will depend on the specific system under study. Our choices were determined by visualising the histograms of each data type which can be found in our supplemental information online (https://github.com/ArianeMora/SiRCle_multiomics_integration). The regulatory labels from RG method 2 were chosen (Table 2, Table S2). ORA (over representation analysis) was performed on each of the clusters using the background as defined by the background method (meaning all genes with *p* < 0.05 in at least two datasets). State unchanged includes unmeasured; for example, a protein may be changed, but not detected. Similarly, given DNA methylation is array-based data, a gene’s initial point of dysregulation may be at the methylation layer, yet not probed.

### TF analysis

TFs were identified in each regulatory cluster using the TF database from DoRothEA [55] (level A, B and C relationships). Relationships were retained only where the TF and the target had a significant (*p* < 0.05) change on the mRNA (target) level, and the direction of change agreed with the direction of mode of regulation (MOR). sciMoTF (version 0.1.1, https://github.com/ArianeMora/scimotf) was used to identify and visualise TFs that were enriched in clusters. Fisher’s exact test (FET) with Benjamini-Hochberg (BH) correction was used, whilst the background consisted of all identified TFs in other regulatory clusters.

### ChIP-seq visualisations

ChIP-seq peaks from HIF1A (GSM3417826, GSM3417827, GSM2723878) were downloaded using ChipAtlas [56] with a threshold of 50 (i.e. for peaks to be present they must have a *Q* value < 1E − 05). These peaks were used to check whether HIF1A peaks did indeed occur at the TSS of the genes in patient derived renal cell carcinoma cell lines. IGV jupyter [57] was used to view the peaks.

### Variational autoencoder to compress patient’s genes’ features

A variational autoencoder (VAE) is a generative machine learning method that can be used to learn to create a latent (i.e. compressed) representation of data. We choose a standard Gaussian as the prior distribution for the latent variables. The conditional distribution of the latent variables given the input data is given by the encoding ${q}_{\theta }(z|x)$, and the conditional distribution of the data given the latent variables is represented by the decoding function ${P}_{\phi }(x|z)$, where the parameters, $\theta$ and $\phi$, include the weights and biases of the neural network that are learnt during training, via optimising with respect to an objective function. The objective function is used to (1) ensure the latent distribution approximates a standard Gaussian by minimising the distance via maximum mean discrepancy ${D}_{MMD}$ and (2) ensure the decoded data point is as close to the input as possible via mean squared error (MSE), i.e. we have a good generative model [73].

$${D}_{MMD}({q}_{\theta }||{p}_{\phi })={\mathbb{E}}_{{p}_{\phi }(z), {p}_{\phi }(z')}[k(z, z^{\prime})]-2{\mathbb{E}}_{{q}_{\theta }(z),{p}_{\phi }(z{\prime})}[k(z, z^{\prime})]+{\mathbb{E}}_{{q}_{\theta }(z), {q}_{\theta }(z')}[k(z, z^{\prime})]$$

Above, the distance between the learnt distribution and the expected distribution is computed via the distance between distributions ${q}_{\theta }$ and ${p}_{\phi }$ using a kernel function, *k*. In our implementation, we use a Gaussian kernel, following the implementation from Zhao et al. [58].

$$\mathrm{MSE}=\frac1n\sum\nolimits_{\mathrm i}^n\left(x_{ip}-\overline x\right)^2$$

MSE is used to calculate the mean difference between the input data point, ${x}_{ip}$, and the decoded value, ${x}_{ip}{\prime}$, where ${x}_{ip}$ is a set of features belonging to one patient, *p*, and one gene, *i*. Features selected as input were protein and mRNA, both tumour normal and difference values, and methylation tumour versus normal difference, equating to a vector of seven features. Given the differing ranges of each feature, the data are scaled w.r.t. gene *i* for a given data type prior to calculating the difference between tumour and normal. For example, we normalise ${y}_{ip}$ as follows: *y* is the value for, e.g. mRNA, *i* is the gene index, and *p* is the patient index, where *min* and *max* are calculated across all patients for gene *i*, omitting any missing data.

$$y_{ip\ normalised}=\frac{y_{ip}-{y_i}_{min}}{{y_i}_{max}-{y_i}_{min}}$$

Prior to selecting the training set, genes with any feature with a *z*-score greater than two are considered outliers and omitted from the training set, however, are still input into the trained model.

Our formulation lends to using the integrated value, ${z}_{ip}$, as input to statistical functions such as Mann–Whitney *U*, or a *t*-test. As an example, we show the formulation of the *t*-statistic on using integrated values, ${z}_{ip}$, as defined in the results section. The test statistic for the difference between two patient groups, *S* and *G*, is thus defined as follows:

$${t}_{i}=\frac{{\overline{z} }_{iS}-{\overline{z} }_{iG}}{\sqrt{\frac{{s}_{iS}^{2}}{{n}_{\left|S\right|}}+\frac{{s}_{iG}^{2}}{{n}_{\left|G\right|}}}}$$

Significance is determined via two-sided null hypothesis, namely that there is no difference between the means of the encoded values for patients belonging to group *S* and group *G*. In the non-parametric case, we use Mann–Whitney *U*, where the null hypothesis that there is an equal chance that a patient from *S*, is greater than a patient from *G* and vice versa, as calculated by ranking each patient via their encoding.

### Creating the integrated dataset

To identify genes that changed significantly across the three data types between patient groups, we developed an integration package using a VAE (formalised above) to first integrate the data, reducing the features across patients to a single data point then perform statistics across two groups of patients. Missing normal data were substituted with the mean normal value from all patients. Only patients with matching protein and RNA normal and tumour data were used for training (*N* = 59). We chose seven features as input to the VAE, specifically, the ${\text{log}}_{2}FC$ for RNA and protein, the methylation difference (beta value) for DNA-methylation, and the individual values used to calculate the ${\text{log}}_{2}FC$ for the normal RNA, tumour RNA, normal protein and tumour protein. We chose to use the ${\text{log}}_{2}FC$ in addition to the normalised values for RNA and protein as the baseline level of RNA and protein provides additional information that is not contained within the ${\text{log}}_{2}FC$. Specifically, a lowly expressing gene may be a TF and as such there is biologically relevant information behind the expression value. We opted to not include the baseline DNA methylation values as they are highly variable and hence may bias the VAE, which we found to favour this data type when included obscuring the other signals (e.g. DNA methylation). We used the data output from the SiRCle clusters as the input data to the VAE, meaning the genes from the SiRCle clusters were used as training samples with each cluster being used to train a different VAE. The choice to train a different VAE for each cluster was motivated by the desire to capture within class variation and ensure that variation in smaller SiRCle clusters such as MDE and MDS were not omitted.

Data (genes by patients) from each SiRCle cluster were used to train a VAE (train-test split = 75:25), with 1 internal layer (5 nodes), and a single latent node, using a maximum mean discrepancy parameter of 0.1, mean squared error loss, rectified linear unit activation functions, Adam optimiser, batch size of 16, maximum epochs of 100 with early stopping after three epochs of no improvement in loss. These configurations were constant for all VAEs, and we end up with 10 trained VAEs corresponding to the 10 SiRCle clusters.

### Statistics on the integrated dataset

To determine genes that changed significantly between the groups of patients across the levels of information, we tested each gene using Mann–Whitney *U* (given MDS_TMDE, TPDS_TMDE, MDE, MDE_TMDS, TMDS and TPDE_TMDS were not normally distributed) on a single comparison across the two groups: early (stage I and stage II) vs late (stage III and stage IV) (Table S1, Additional file 1: Fig. S1a). *p* values were corrected using BH FDR (Benjamini–Hochberg false discovery rate) on all genes within each cluster. The latent feature of the VAE can be thought of as the integrated change for a specific gene across the seven input features to the VAE. Thus, the difference between the two cohorts is the mean integrated difference across a gene between the two patient groups.

Before returning the values to the user the direction of the VAE integrated value with that of protein log_2_*FC*, or RNA log_2_*FC* (Additional file 1: Fig. S5a). To quantify the integrated change, termed “integrated difference”, we computed the “distance” condition 1 (e.g. late stage) patients’ mean VAE value from condition 0 (e.g. early stage) mean VAE value (subtracting the value from condition 0 from condition 1 in an absolute sense). Note this is defined earlier.

We used fGSEA test (fast gene set enrichment analysis) [59] to identify coordinated changes in metabolic signatures [60] and pathways using the integrated difference as the statistic provided to fGSEA and use 10,000 permutations.

### Metabolomics profile comparing early and late stage

Median normalised metabolomic profiling data of 138 matched clear cell renal cell carcinoma (ccRCC) and normal tissue pairs was downloaded from Table S2 of Hakimi et al. [7]. Tumour samples were normalised to their matching normal tissue sample based on the matching index by calculating the fold change. Next, we performed differential metabolite analysis (DMA) comparing late stage (stage III and IV) versus early stage (stage I and II) and old (age at surgery > 58) versus young (age at surgery < 42) patients. Mann–Whitney *U* test (wilcox.test in R) was used to calculate the *p* value and corrected using BH. Unnamed metabolites were removed, and metabolic pathways assigned using published pathway information [7].

### *ORA* overlaps

Overlapping the ORA results was performed for each cluster separately, GO terms with greater than 10 genes associated were retained and with a *p*.adj value < 0.05. These significant terms were overlapped to the original ccRCC cohort results.

## Results

### Functional effects of ccRCC are regulatory layer dependent

ccRCC is a heterogeneous cancer with transformation and progression linked to widespread dysregulation of biological processes. To understand how cellular processes are affected, analyses must extend across regulatory layers and incorporate multiple omics’ data types. Recently, Clark et al. and the CPTAC consortium investigated the impact of genomic alterations on protein regulation and created revised subtypes based on integrated analyses [14]. As part of their study, Clark et al. generated protein, RNA and DNA methylation measurements from 110 patients with ccRCC [14], of which we filter to 76 cases, see “Methods” for details.

We sought to investigate the compounding effects of dysregulation by following gene changes from the DNA methylation via the mRNA to protein layers to unravel the regulatory layer of each gene within ccRCC. The original study by Clark et al. did not include normal methylation samples thus our study extends their work by also considering the effect of epigenetic changes in the context of ccRCC (see Table S1 for sample annotations and “Methods” for quality controls). The CPTAC cohort is demographically homogenous but includes cases across four stages of ccRCC and multiple mutational patterns (Table S1, Additional file 1: Fig. S1a). Data types are herein denoted “data layer” as they correspond to distinct layers of regulation (DNA methylation beta values, normalised mRNA expression and normalised protein expression). PCA emphasises the primary source of variation in each data layer is the sample type (tumour versus normal) with neither of the top two components explaining tumour stage (Additional file 1: Fig. S1a).

**Fig. 1:**
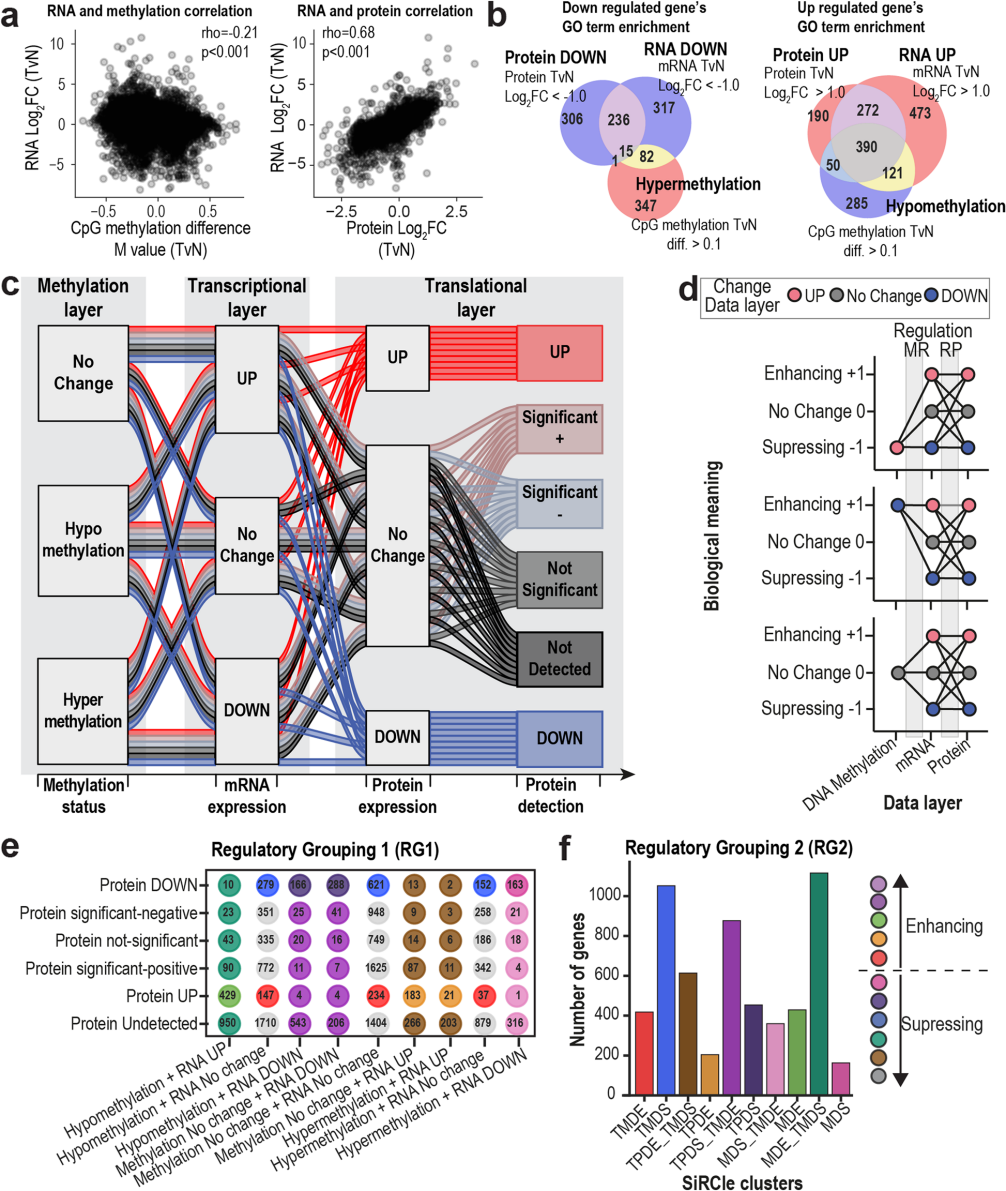
SiRCle model logical regulatory rules. **a** Spearman correlation comparing the change between tumour and normal samples for mRNA expression and DNA methylation, and mRNA expression and protein expression. **b** Numbers of enriched Gene Ontology (GO) terms when comparing tumour versus normal (TvN) for each data type, indicating shared terms by overlapping regions in the Venn diagram. “Down regulated” corresponds to GO terms enriched in genes that are decreased in tumour samples, whilst “Up regulated” is for GO terms enriched in genes increased in tumour samples. **c** Alluvial plot depicting the flows used to define SiRCle clusters. The plot is read from the left to the right, in line with the flow of information in a biological system. Each data type has been labelled as a layer, with one of three states defined for each layer based on the results for differential analysis between tumour versus normal in that data type. The “No Change” on the translational layer is further subdivided to reflect protein detection and threshold criteria met. **d** Explanation of the directions of change and the corresponding biological meaning for each layer. **e** Summary of collapse of regulatory groupings coloured by regulatory grouping 2 which is used throughout the paper. **f** How the ccRCC data is dispersed into the different regulatory clusters using the SiRCle grouping method with the biological outcome described on the side

We identified genes changing significantly between tumour and normal by performing differential analysis tests for each data layer and found widespread changes, most notably on the mRNA layer (“Methods”, Table S1). Whilst there is minimal negative correlation between gene changes on the DNA methylation and mRNA layers, the mRNA and protein layers are correlated (Fig. 1a). We found 1284 significant genes shared across the mRNA and protein layers and 796 significant genes between all three layers, where significant is defined as *p* < 0.05, |beta_diff|> 0.1, |protein_logFC|> 0.5 and |rna_logFC|> 1.0.

Given the small number of significant genes shared across layers, yet comprehensive changes within each layer, we sought to determine whether the affected genes shared biological function. To do this, we performed over representation analysis (ORA) of Gene Ontology (GO) terms on significant genes from each differential analysis, sub-setting genes by direction of change. We observed both unique and shared biological functions between the layers, with more similar functions enriched across layers for upregulated than downregulated genes (Fig. 1b). Hypermethylated genes were most enriched for terms associated with development (methylation layer), whilst supressed transcripts were most associated with transporter activity (mRNA layer) and supressed proteins with mitochondrial processes (protein layer) (Additional file 1: Fig. S1b–c, Table S2). In line with the findings from the original study [14], immune response terms showed the most significant enrichment across all data layers for genes upregulated on the protein and mRNA layers (Additional file 1: Fig. S1b–c, Table S2).

### Following the flow of information extracts clusters that drive specific cellular phenotypes in ccRCC patients

Given the lack of correlation between the data layers and the heterogeneity of functional enrichment, we posited that grouping genes by their pattern across layers, prior to performing ORA would facilitate biological interpretation. Based on the differential analysis, we defined three states comparing tumour and normal for DNA methylation, mRNA expression and protein abundance, namely positive, negative or unchanged, for each gene and layer (Fig. 1c, Table 1, “Methods” section). Given that the protein abundance is the data layer closest to the phenotype, yet at the same time has the lowest coverage we aim to account for this. Hence, “No Change”, which includes proteins that do not meet the significance and/or the fold change threshold, is subdivided into four states for the protein layer: 1. “undetected” (genes detected in other data layers, but not in the proteomics data layer), 2. “not significant” (proteins that do not meet the significance threshold), 3. “significant negative” and 4. “significant positive” (proteins that do not meet the negative/positive fold change threshold but meet the significance threshold) (Fig. 1c, labelled, translational layer). Since this is an ordered series of three 3-state-3-state-6-state transitions between the layers, there are 54 possible “flows”. Each gene will be assigned to one of those 54 “flows”, which is required to enable the summary of genes into clusters.

To perform biologically meaningful clustering, we group genes based on the layer where the gene “changed” in the tumour sample compared to the normal sample and termed this “Regulation Grouping” (RG). We do this by following the central dogma of biology [61], i.e. genetic information proceeds from DNA, to mRNA, then to protein (Fig. 1c–d). For example, if a gene is hypermethylated, has a decrease in mRNA expression and displays a decrease in protein expression, we can likely conclude that the dysregulation first occurred on the DNA methylation layer, meaning this gene is supressed via Methylation-Driven Suppression (MDS). Whilst there are 54 possible flows, we use the RG to summarise these 54 flows into SiRCle clusters based on combinations of regulations between DNA methylation and mRNA expression (MR) and/or between mRNA expression and protein expression (RP) (Fig. 1d–e), ultimately defining the SiRCle cluster names that reflect those regulations (“Methods”, Table 1).

We term the process “**Si**gnature **R**egulatory **Cl**ust**e**ring” (SiRCle), and throughout the paper, we use “RG2_Change” with ten clusters (Fig. 1f). As an input for SiRCle clustering, we include genes that are either detected on the protein layer or on both the mRNA and DNA methylation layers (background P¦R&M). For more information on thresholds, RG and background see “Methods” and the package guidelines.

**Fig. 2:**
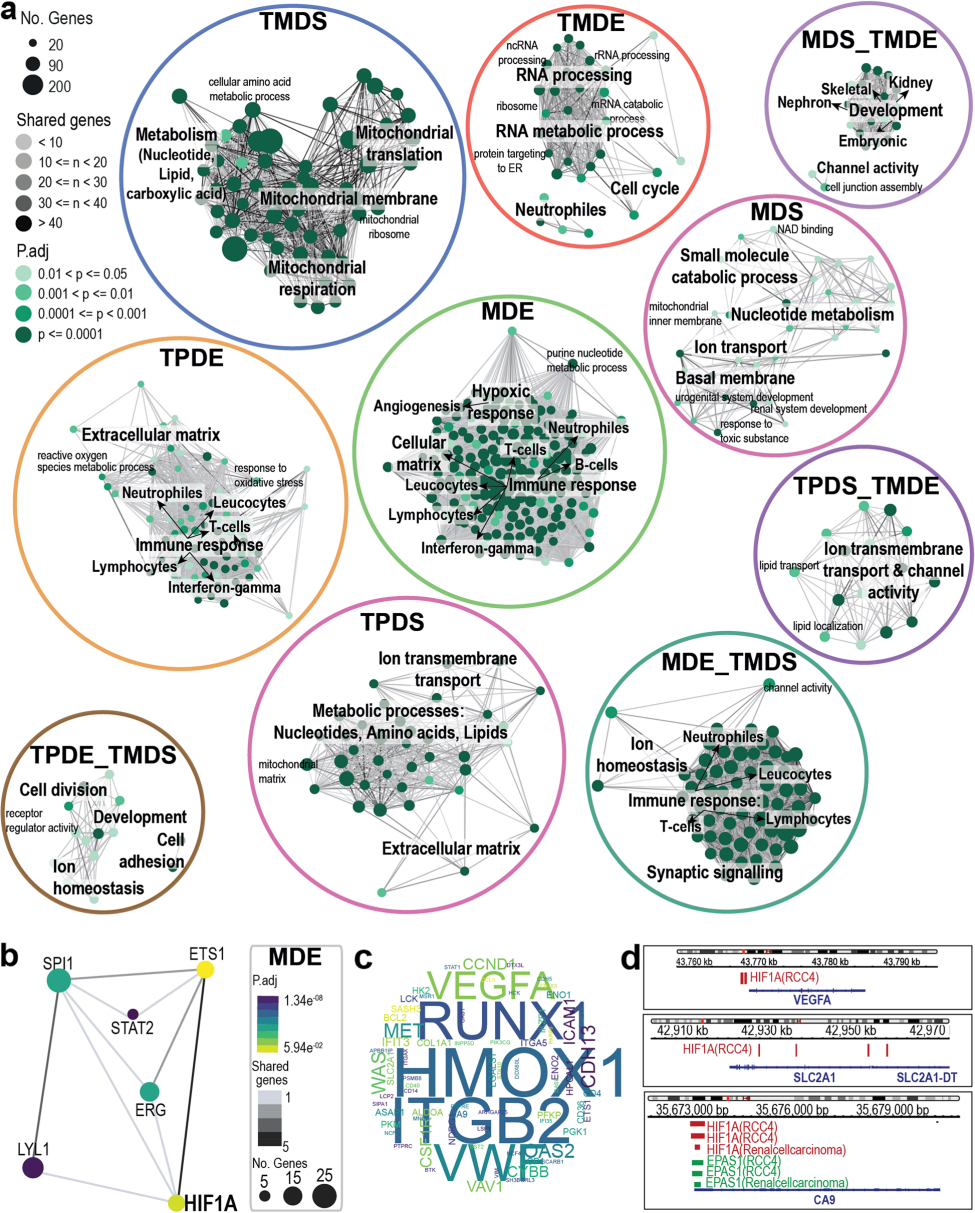
Phenotypic changes in ccRCC and TF drivers. **a** Emapplot visualisation of the over representation analysis (ORA) performed on each SiRCle cluster resulted in biological pathways that are altered comparing tumour versus normal. Pathways were plotted if *p*-adjusted value (*p*.adj) < 0.05 and the gene ratio was greater than 5%. The dot size corresponds to the number of genes found in the cluster that are part of the biological pathway. The colour of the dot shows the *p*.adj of the ORA. The connecting lines (grey) show that the biological pathways have genes in common. **b** Transcription factor (TF) network of TFs from manually curated repositories (DoRothEA) that may drive genes in the cluster Methylation Driven Enhancement (MDE). The dot size corresponds to the number of genes targeted by the TF. The colour of the dot shows the *p*.adj. The connecting lines (grey) show the number of common genes the connected TFs regulate. **c** Wordcloud including the TF targets with shared TF binding in the MDE cluster, with the size corresponding to how many different TFs are predicted to regulate the gene’s expression. **d** HIF1A ChIP-seq peaks (RCC4, GSM3417827) binding at the transcription start site of *VEGFA*, *SLC2A1* and *CA9*

Next, we performed ORA on the SiRCle clusters (Fig. 1f) and found that each cluster was enriched for a biological signature important in ccRCC and consolidated the functional enrichment results we observed across each layer when independently analysed (Fig. 2a, Table S2). We found clear biological signatures for both SiRCle clusters regulated on one layer and SiRCle clusters regulated on multiple layers. Specifically, hypomethylation (Methylation-Driven Enhancement (MDE)) was associated with the hypoxic response, oxidative stress response and angiogenesis (Fig. 2a), key players in ccRCC tumours [46]. These terms were not revealed in the top terms when we performed ORA on each layer independently (Additional file 1: Fig. S1b–c).

In line with previous findings [14, 46, 62], we observed an increase in immune response pathways and show these genes are likely regulated by DNA hypomethylation (MDE, Fig. 2a) or enhanced transcription (Transcription and Processing-Driven Enhancement (TPDE), Fig. 2a). Clark et al. described a high correlation between mRNA and protein expression of immune signatures [14], supporting our finding that the regulation occurs at the transcriptional layer. We were also able to distinguish between immune response genes likely regulated by hypomethylation from those likely regulated by enhanced transcription (Fig. 2a).

We observed that genes in two SiRCle clusters are involved in distinct metabolic rewiring, with metabolic processes such as lipid and amino acid metabolism likely regulated by transcriptional suppression (Transcriptional and Processing-Driven Suppression (TPDS)), whilst mitochondrial respiration and nucleotide metabolism are likely downregulated by translational suppression of the proteins (Translation and translational-modification-Driven Suppression (TMDS), Fig. 2a). Genes regulated by translational suppression appear to be involved in mitochondrial metabolism, mitochondrial translation and mitochondrial morphology (TMDS, Fig. 2a). Hypermethylated genes and/or translationally enhanced genes were enriched for kidney development, hinting towards a loss of cellular identity in cancer cells (MDS_TMDE, Fig. 2a). Using SiRCle clustering, we were able to determine the layer at which a gene’s dysregulation occurs and find clusters corresponding to distinct biological processes that may underpin tumour pathology.

### Transcription factors as drivers of genes in SiRCle clusters

A subset of SiRCle clusters contain genes that change their state at the transcriptional (TPDE/TPDS) and/or the methylation layer (MDE/MDS), suggesting that they are regulated by transcription or epigenetic factors. Indeed, a specific transcription factor (TF) may drive such changes in gene expression as TFs can act to enhance or repress gene transcription and DNA methylation changes can alter the TF’s ability to bind to the DNA [63]. We used validated TF-to-target interactions from DoRothEA [55] to identify which TFs were statistically associated with each SiRCle cluster. We use Fisher’s exact test (FET) to measure association between a TF and SiRCle clusters by testing if a TF targets more genes in a single cluster relative to the background frequency (all SiRCle clusters). This analysis only recovered TFs significantly associated with SiRCle clusters that are either up- or downregulated at the methylation and transcriptional layers (Fig. 2b–c, Table S3). The stratification provided via SiRCle clustering helps distil the data from which regulatory logic can be inferred.

Given that HIF1 drives angiogenesis in ccRCC tumours [46], we were unsurprised to find HIF1 TFs target genes in MDE as this cluster was enriched for angiogenesis related GO terms (Fig. 2a–b, Additional file 1: Fig. S2a, Table S3). We found that HIF1A had a significant increase in target-mRNAs driven by greater accessibility to binding due to DNA hypomethylation, which also translates to a detectable change on the protein layer (MDE). Given DoRothEA is not tissue specific, we sought to confirm that the HIF TFs bind to the target genes in ccRCC by using ChIP-seq data from HIF1A and EPAS1 (also known as HIF2A) in kidney cancer cell lines (see “Methods”). We found evidence of HIF1A binding at the transcription start site (TSS) of genes in MDE (*VEGFA*, *CA9* and *SLC2A1*) (Fig. 2d) including many metabolic enzymes (*HK2*, *ALDOC*, *GAPDH*, *PKM*, *ENO1*, *ENO2* and *LDHA*, Additional file 1: Fig. S2a). Our findings support that TF analyses coupled with SiRCle can elucidate relationships at both the transcriptional and methylation layers enabling the identification of novel TF targets (Fig. 2b, Additional file 1: Fig. S2c), which can be investigated for changes in downstream products and/or be followed up experimentally.

### ccRCC characteristic metabolic changes are detected on distinct layers

**Fig. 3:**
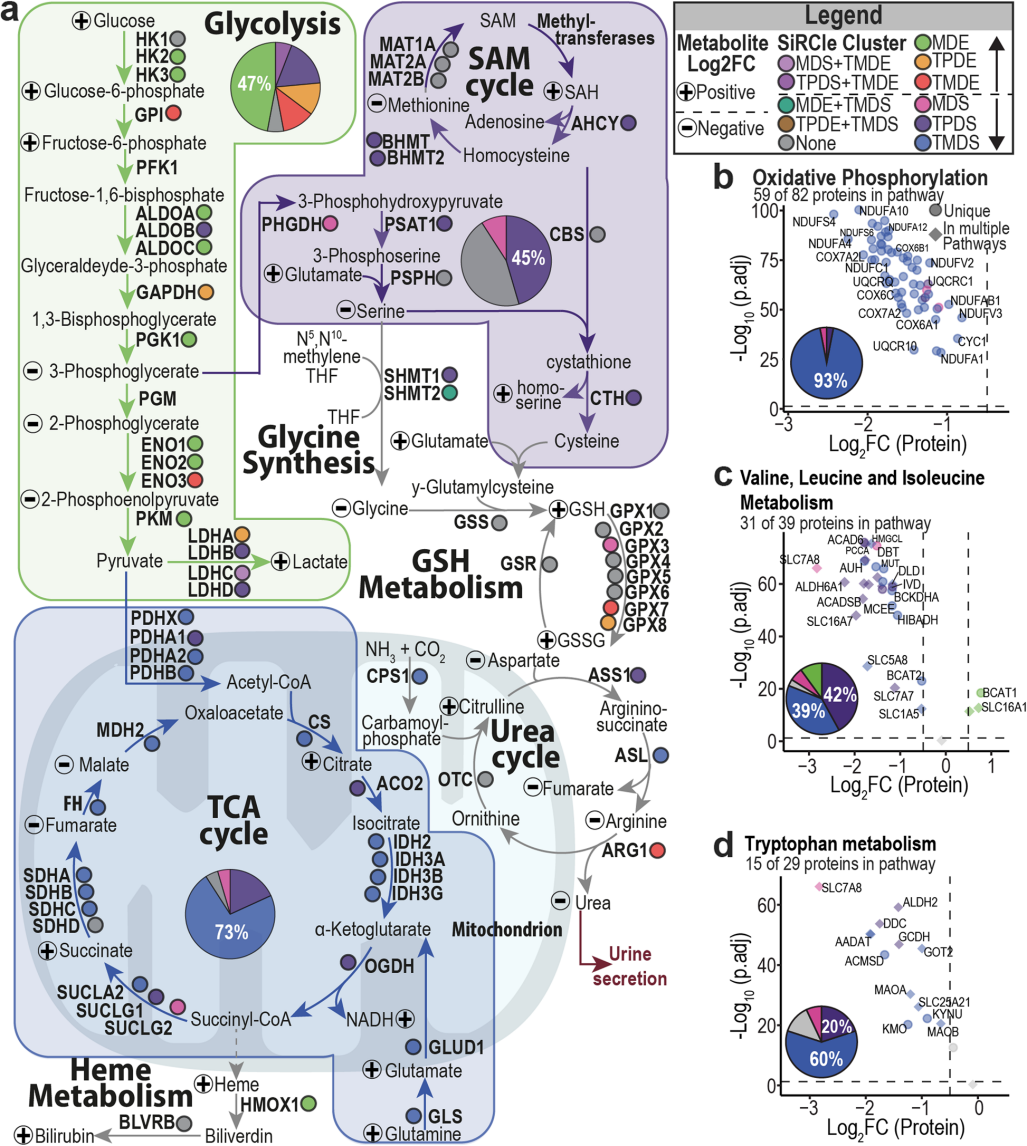
Metabolic changes in tumour samples are regulated on distinct data landscapes. **a** Metabolic changes of glycolysis, tricarboxylic acid (TCA) cycle and serine, glycine and cysteine biosynthesis in ccRCC comparing tumour versus normal. Metabolic enzymes are written in bold and if they have been detected, they are labelled with coloured circles depending to the SiRCle cluster they are part of. Metabolite changes are labelled with plus for a positive and minus for a negative log_2_*FC*. The three main metabolic sections, categorized based on the SiRCle cluster to which the majority of metabolic enzymes belong, are summarised in pie charts. **b**–**d** Volcano plots are based on the metabolic pathways defined by Gaude and Frezza [60] and the number of proteins detected within the pathway is reported. Proteins that are unique for the metabolic pathways are displayed as circles and proteins that are part of multiple metabolic pathways are displayed as diamonds. The colour code is based on the SiRCle cluster the protein is part of and is summarised in the pie chart. GSEA was performed using the protein statistic. Here, we plot oxidative phosphorylation (**b**), valine, leucine and isoleucine metabolism (**c**) and tryptophan metabolism (**d**), all reported a GSEA *p*.adj of 0.000966 (the lowest *p*.adj by threshold limits)

Metabolic rewiring plays a crucial role in ccRCC and hence we sought to investigate the dysregulation of metabolic genes to understand the layer at which the metabolic pathways are altered. We performed gene set enrichment analysis (GSEA) using metabolic signatures from Gaude and Frezza [60], herein referred to as metabolic signatures, to identify pathways with coordinated protein changes in tumour versus normal. The protein layer was chosen for GSEA to capture changes in pathways’ enzyme activity, and then coupled with SiRCle cluster annotations to relate gene changes to the initial layer of dysregulation (Table S4). We found the majority of enzymes involved in glycolysis (~ 70%) were increased comparing tumours versus normal, fitting with the previously established upregulation of anaerobic glycolysis in ccRCC [8]. Interestingly we find that 47% of glycolytic enzymes are hypomethylated and upregulated on both the mRNA and protein layers (SiRCle cluster MDE, Fig. 3a and Additional file 1: Fig. S2b–c). From the TF analysis, we noticed that HIF1A targets in MDE included several glycolytic enzymes, and we also found HIF1A binding at the transcription start site of glycolytic genes in kidney cancer cell line ChIP-seq data (Additional file 1: Fig. S2c).

We next sought to dissect the role of mitochondrial dysfunction in ccRCC, including the suppression of mitochondrial electron transport chain (ETC), since this has been previously reported [8] to play a role in ccRCC, yet how metabolic enzymes are regulated remains unclear. We found that enzymes in electron transport chain complexes were depleted in the tumour samples and were likely regulated at the translational layer (SiRCle cluster TMDS, Fig. 3b). ETC includes genes encoded by the nuclear genome regulated on the translational layer (TMDS), whilst genes encoded in the mitochondrial DNA were found in the transcriptional regulation cluster (TPDS_TMDE). Yet, given that we do not include any normalisation for mitochondrial copy number, this could change once the mitochondrial copy number is taken into account. Hence, we propose that decreased genomic translation leads to instability of the complexes and protein degradation and vice versa. The translation-driven downregulation affects almost all enzymes of the TCA cycle (SiRCle cluster TMDS, Fig. 3a and Additional file 1: Fig. S2d). In addition to these primary metabolic rewiring steps, there are many secondary pathways altered in ccRCC [8]. By coupling SiRCle annotations with GSEA, we found that the suppression of cysteine methionine and glutathione (GSH) metabolism [7] occur on the transcriptional layer (SiRCle cluster TPDS, Fig. 3a and Additional file 1: Fig. S2e, f). Similarly, serine biosynthesis, which is important to fuel GSH biosynthesis, also occurs on the transcriptional layer. In accordance with the ORA results (Fig. 2a), we found that other amino acids’ metabolic pathways such as tryptophan, valine, leucine and isoleucine are downregulated either by translational or transcriptional suppression (SiRCle clusters TPDS and TMDS, Fig. 3c–d).

To understand the impact of the enzyme expression on the metabolic profile, we used metabolomics data from 84 ccRCC patients with paired tumour and adjacent tissue samples [7] and performed differential metabolomics analysis to identify the metabolites significantly changing between tumour and adjacent tissue. To consolidate the metabolite levels with enzyme information, we assigned significant metabolite changes to the pathways identified from the protein layer (Fig. 3a). We observed a “split” of glycolysis as previously described by Hakimi et al. [7], whereby metabolites upstream of GADPH are accumulated, and the downstream metabolites are depleted, despite the majority of glycolytic enzymes being upregulated on the protein layer (Fig. 3a, Additional file 1: Fig. S2b, Table S4). A similar split is observed in the TCA cycle, where citrate and succinate are increased despite a decrease in the enzyme’s protein expression, whilst fumarate and malate are depleted in line with the enzyme’s decreased protein expression (Fig. 3a). Overall, we show that SiRCle clustering can elucidate the layer where metabolic changes in ccRCC are orchestrated, highlighting points of intervention for targeting metabolism as anticancer strategy.

### Functional differences between late and early-stage tumours show limited agreement across layers

**Fig. 4:**
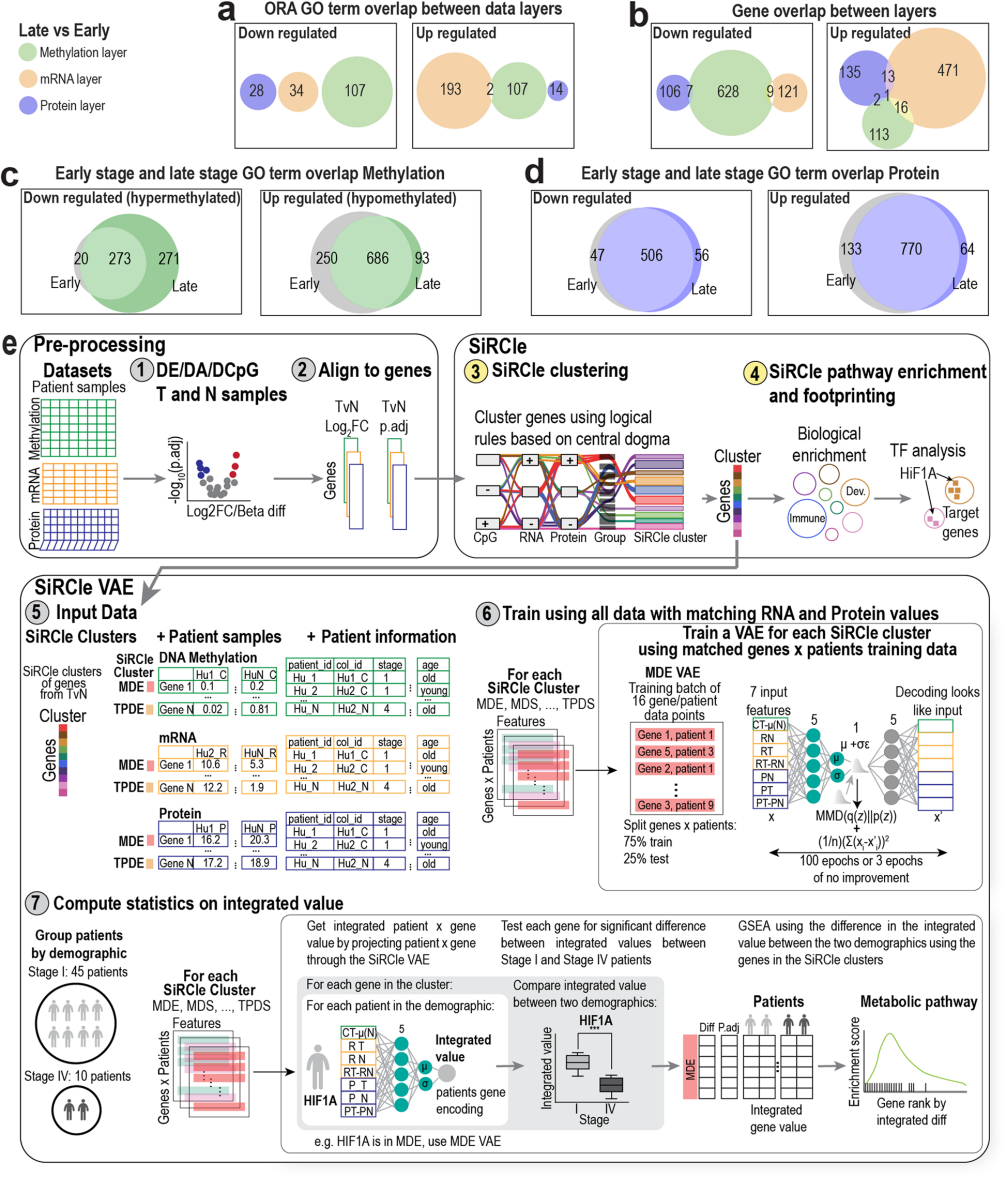
Late vs early tumours data integration. **a** Overlap of GO terms from ORA performed on each layer separately using the significant GO terms from differential analysis comparing late-stage and early-stage tumours. **b** As in **a**, except the gene overlap not the GO overlap. **c** Overlap of GO terms from ORA on DNA methylation for tumour vs normal on early-stage and late-stage datasets independently. **d** As in **c** except using the protein layer. **e** Overview of the SiRCle approach from the pre-processing (up to user), and SiRCle clustering, to the SiRCle integration for post hoc analyses

The previous sections explored dysregulation in tumour samples across a heterogeneous cohort of ccRCC patients. However, it has been reported that tumour stage is an important determinant of prognosis and treatment response [45]. We thus sought to identify the regulatory differences between patients with early-stage (stage I, *N* = 30 and stage 2, *N* = 8) and late-stage (stage III, *N* = 27 and stage IV, *N* = 11) cancer by performing differential analysis on each layer, between tumour versus normal, for each patient group independently (Table S5). Using the significant genes from each analysis, we performed ORA on each data layer (Fig. 4a-b). On the protein and mRNA layers, we found that similar biological terms were enriched across the two patient’s groups, whilst on the methylation layer there were far more terms enriched in the late-stage samples (271) (Fig. 4c–d). The main differences between the top associated GO terms were an association with the regulation of lymphocytes (hypomethylated) and DNA-binding transcription activator activity (hypermethylated) in late-stage samples (Additional file 1: Fig. S3a).

It is unsurprising that the tumour versus normal comparisons yielded similar results despite being run on patients with different stage tumours as the primary source of variation in the dataset is accounted for by the sample type. Hence, we next tested for differences in tumour profiles between late (stage III and IV) and early-stage (stage I and II) tumour samples (Table S5). When comparing late versus early-stage tumours, we found few changes on the protein layer, but aberrant changes on the DNA methylation and mRNA layers (Fig. 4a–b, Additional file 1: Fig. S3b). As tumours progress, the dynamic adaptability of metabolism plays a crucial role to ensure cell growth [64], and therefore, we tested for coordinated changes across metabolic signatures using the differences between late and early-stage tumours. We found minimal shared enrichment (*P*_*corrected*_ < 0.05) of pathways between layers, with only protein modification enriched on all three layers (Table S5). Whilst we find significantly changing metabolic pathways within each layer, functional information is not readily captured across layers rendering the results challenging to interpret. However, we note that given the hierarchy of GO terms, there is a possibility that the layers have higher shared function via overlapping parental terms not captured by a direct comparison.

### Integrated statistical test to identify changes between patient cohorts

Given the independently performed differential analyses were unable to extract shared functions when comparing patients with late versus early-stage tumours, we posited that integrating across the data layers prior to performing differential analysis and biological enrichment may better capture biological signal. We opted to use a VAE to learn gene-wise relationships across the three data layers for each patient, resulting in an integrated value for each patient enabling analysis between patient groups on, e.g. stage difference (Fig. 4e). Akin to matrix factorisation methods, a VAE finds a projection of data that centres on variance. However, unlike linear methods, such as PCA, a VAE does this using a neural network and can thus capture non-linear relationships.

In short, there are two parts of a VAE, (1) an encoding function that transforms data to a compressed representation and (2) a decoding function that recreates the input from the compressed representation. The encoding and decoding functions are shared once the parameters have been learnt from the training data, whilst an encoded value is specific to a given data point. For example, the compressed representation of data point ${x}_{ip}$ is given by ${{z}_{ip} \cong q}_{\theta }\left(z|{x}_{ip}\right)\cong {\mu }_{ip}+{\sigma }_{ip}\odot {\epsilon }_{l}$, where ${z}_{ip}$ is the latent representation produced by the model conditioned on ${x}_{ip}$, ${q}_{\theta }$ is the encoding function, ${\epsilon }_{l}$ is stochastic noise and part of regularisation, ${\mu }_{ip}$ is the encoded mean and ${\sigma }_{ip}$ is the encoded variance. Note, ${x}_{ip}$ refers to a feature vector of a gene, denoted by index $i$ for a specific patient $p$. The feature vector is the normalised values across the data layers (Fig. 4e).

The encoding and decoding functions are “learnt” by minimising an objective lower bound over a dataset, by iteratively updating parameters during training. For our purposes, the objective is to minimise the difference between input and output as per mean squared error and maximise the similarity between the latent space and a Gaussian normal distribution via maximum mean discrepancy (see “Methods” for specifics).

Given the relatively low signal produced by stage, extracting regulatory variations may be obstructed by the noise in the dataset when considering all regulatory relationships at once. As such, instead of learning a dataset-wide representation, we used genes from each SiRCle cluster to learn a representation that defines a given regulatory flow. As such, there is an encoding function ${q}_{\theta }\left(z|x\right)$ for each SiRCle cluster.

As we can now calculate an integrated (encoded) value for each gene, for each patient, we can define a gene’s integrated difference as a gene’s mean encoding difference between two sets of patients, e.g. patients with late versus early tumours (Fig. 4e).

Mathematically, we define this below, where *z* refers to the latent encoding given a particular data point, which corresponds to a patient’s gene value across the layers. Gene index $i$ is held constant as the mean difference is calculated between patient sets, for example patients with a late tumour may be in set *S*, and those with an early tumour are in set *G*.

$$D{(z}_{iS}||{z}_{iG})=\frac{1}{\left|S\right|}{\sum }_{s=1}^{\left|S\right|}{z}_{is}-\frac{1}{\left|G\right|}{\sum }_{g=1}^{\left|G\right|}{z}_{ig}={\overline{z} }_{iS}-{\overline{z} }_{iG}$$

For biological interpretation, we can correlate the integrated difference to a biological reference such as the mean difference between the two patient sets on the protein layer. For example, in the MDS SiRCle cluster, we see that the late versus early stage integrated difference correlates to the difference on the DNA methylation, protein and mRNA layers between these patient sets, whilst TPDE does not seem to be affected by DNA methylation, as expected (Additional file 1: Fig. S4a).

Using our integrated value for each gene, we next perform a Mann–Whitney *U* test to identify genes with a significant integrated difference between patients with early-stage (stage I, *N* = 30 and stage 2, *N* = 8) and late-stage (stage III, *N* = 27 and stage IV, *N* = 11) tumours. In the following sections, we demonstrate that VAE integration prior to performing analyses enables us to identify changes across the regulatory layers that were not found when performing differential analyses independently on each data type. Moreover, by benchmarking the VAE’s capacity to capture biological information on the integrated dimensions to six other cancer integration methods (intNMF [33], iCluster [34], JIVE [35], MCIA [36], RGCCA [37], tICA [38]) using the integration benchmarking package MOMIX [17], we find that our VAE approach performs comparatively, if not better at extracting biologically relevant orderings of genes, than existing methods (Additional file 1: Fig. S4b).

### Metabolic alterations in tumour stage impact ccRCC’s metabolic fingerprint

**Fig. 5:**
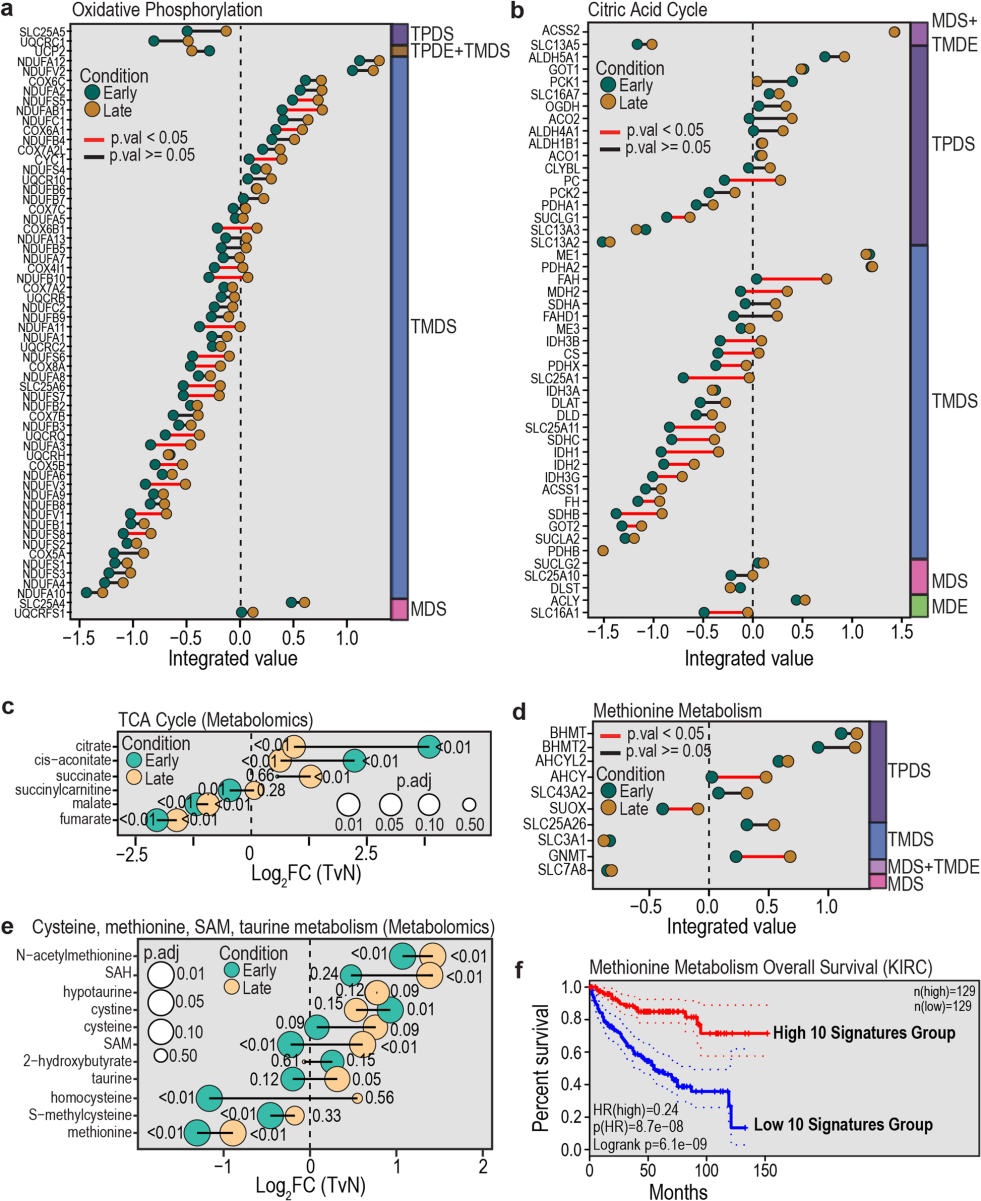
Metabolic alterations in tumour stage impact ccRCC’s metabolic fingerprint. Metabolic pathways based on gene expression are defined by Gaude and Frezza [60] with *p*.adj values corresponding to the GSEA results after ranking the genes in each SiRCle cluster using the VAE integrated rank. Metabolite pathways based on metabolites are defined by Hakimi et al. [7]. **a** Comparison of the VAE integrated rank of late and early-stage patients for oxidative phosphorylation for genes within the Translation and post-transcriptional Modification Driven Suppression (TMDS) SiRCle cluster. **b** Comparison of the VAE integrated rank of late with early patients for TCA cycle genes colour coded for the SiRCle clusters the gene belongs to. **c** Comparison of the ${\text{log}}_{2}FC$ of tumour versus normal (TvN) of late stage (III and IV) with early stage (I and II) patients for TCA cycle metabolites. **d** Comparison of the VAE integrated value of late with early patients for genes corresponding to the methionine metabolism pathway colour coded for the SiRCle clusters the gene belongs to. **e** Comparison of the ${\text{log}}_{2}FC$ (TvN) of late stage (III and IV) with early stage (I and II) patients for “cysteine, methionine SAM, taurine metabolism” metabolites. **f** Methionine survival curve based on (http://gepia2.cancer-pku.cn/) using median default cutoffs of 50%, 50% for ccRCC from TCGA

Whilst there were few shared metabolic pathways in the layer-specific analyses, an integrative model may improve our ability to understand metabolic differences between ccRCC patient groups. As metabolic profiles alter during tumour progression [64], we tested for integrated differences between patients with late and early tumours by performing GSEA to identify pathways with coordinated integrated differences between the two groups (Fig. 4e). We define regulation as “up” if the integrated value increases and as “down” if the integrated value decreases. The integrated value is positively correlated with the mRNA or protein layers, as such it carries biological meaning however, “up” regulation of the integrated value of a specific gene does not equal the same increase in mRNA or protein (Additional file 1: Fig. S4a). In line with previous observations [14], we find oxidative phosphorylation was upregulated when comparing late with early-stage (Fig. 5a, Table S6), yet we are now additionally able to understand on what layer the enzyme expression is defined. We observed that oxidative phosphorylation enzymes are likely altered on the translational layer. Reassuringly, oxidative phosphorylation was also a significant pathway in the late versus early-stage tumour protein layer GSEA analysis, but not on the mRNA layer, matching our TMDS label and showing that trivial single-layer relationships are captured by the VAE approach (Table S6). The coordinated change observed in oxidative phosphorylation was also observed for enzymes of the mitochondrial TCA cycle, with some significantly (*p*.val < 0.05) upregulated enzymes such as *IDH1-3*, *FH* and *SDHB* in late-stage compared to early-stage (Fig. 5b). To understand if this is in accordance with the metabolite levels, we use metabolomics data comparing late (III and IV) with early-stage (I and II) patients published by Hakimi et al. [7]. Interestingly, citrate and cis-aconitate metabolite levels were depleted in late-stage compared to early-stage patients, whilst the downstream metabolites remained unchanged over the stages and have decreased protein expression in the tumour tissue (Fig. 5c).

A novel finding from Hakimi et al. was that metabolites involved in methionine metabolism are altered between different tumour stages in ccRCC [7]. We sought to build on this by investigating whether the changes in methionine metabolism could be traced back to a regulatory change. We found that enzymes of the methionine metabolism pathway are predominantly up on the transcriptional layer when comparing late-stage vs early-stage (Fig. 5d–e, Table S4). *BHMT*, *SLC7A8* and *SLC3A1* were almost unchanged between late and early-stage patients, whilst all other enzymes were up in late-stage patients (Fig. 5d). We observed an overall accumulation of metabolites within this pathway in the late-stage patients, indicating methionine depletion is less severe in late-stage patients (Fig. 4e). It has been recently discussed that enzymes involved in methionine metabolism play a role in ccRCC patients’ survival [65], which we also observe using KIRC (Fig. 5f). Methionine upregulation could increase the methylation potential and hence alter the DNA methylation landscape within a tumour. Given BHMT is a proximal tubule-specific protein and SLC3A1 is a kidney amino acid transporter mainly found in proximal tubule of the kidney, these enzymes may additionally be important for the cellular identity.

Together these results showcase the importance of taking patients’ information into account when analysing and interpreting patient data and that VAE integration enables the comparison of small patient groups whilst considering differences across DNA methylation, mRNA and protein layers.

### SiRCle applied across cancers highlights shared and unique functions with ccRCC

**Fig. 6:**
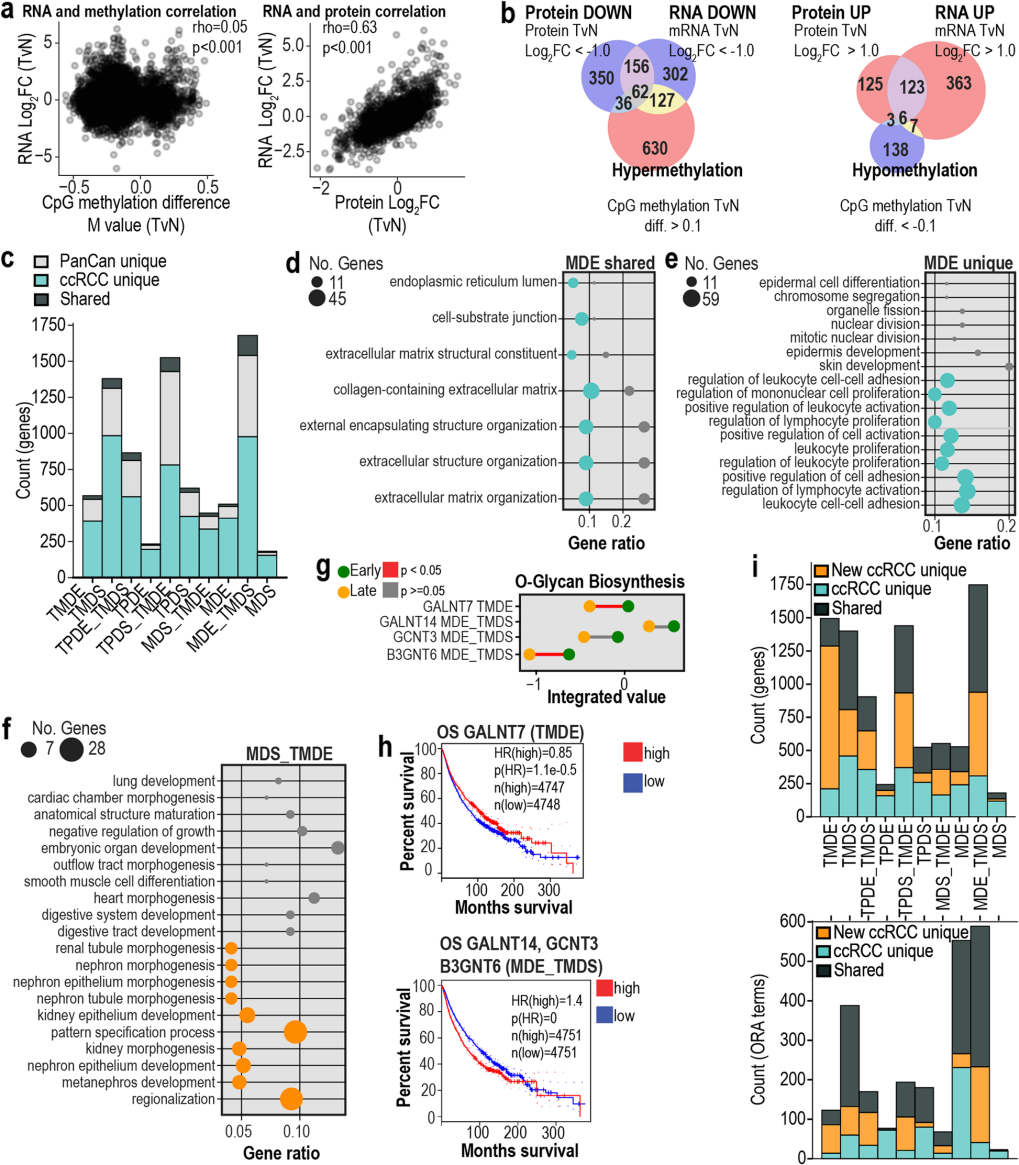
PanCan results. **a** Correlation between DNA methylation and RNA, and RNA and protein for all cancers, where each point represents a gene and the mean change between tumour and normal samples. **b** The overlap between downregulated and upregulated GO terms where GO was performed on each of the data types independently using the annotated cutoffs. **c** Overlap of the genes between the SiRCle clusters from PanCan and ccRCC with shared genes highlighted. **d** Overlap of the top 10 shared GO terms between ccRCC (green) and PanCan (grey) for the Methylation Driven Enhancement (MDE) cluster. **e** Top 10 unique GO terms in ccRCC (green) and PanCan (grey) for the MDE cluster. **f** Integrated value for Methylation Driven Suppression and translational-modification-Driven Enhancement (MDS_TMDE) with ccRCC (orange) and PanCan (grey). **g** O-glycan biosynthesis pathway showing the integrated value using the SiRCle VAE integration. **h** Overall survival from GEPIA using median cutoff for enhanced gene on the protein level (TMDE) and repressed genes on the protein level (MDE_TMDS) for all cancers in TCGA. **i** Overlap of the genes between the SiRCle clusters from ccRCC and the new heterogeneous ccRCC cohort with shared genes (top) and shared ORA terms (bottom) highlighted

To test how SiRCle performs in the context of other cancers, we curated a multi-omic dataset spanning four cancer types, herein termed PanCan. For this, we collected proteomics, transcriptomics and DNA methylation data from 306 patients from CPTAC with corresponding tumour and normal samples, including Head and Neck Squamous Cell Carcinoma (*N* = 55) [48], Lung Adenocarcinoma (*N* = 96) [49], Lung Squamous Cell Carcinoma (*N* = 97) [50] and Pancreatic Ductal Adenocarcinoma (*N* = 58) [51]. Using this PanCan dataset, we performed differential analysis comparing tumour versus normal tissue to extract altered gene regulations that change across all the tissue and cancer types, using disease as a factor. In our PanCan analysis, we found lower gene-wise correlations between mRNA and DNA methylation layers than in ccRCC, but similar correlation on mRNA and protein (Fig. 6a), as in the ccRCC analysis (Fig. 1a). Furthermore, as shown for ccRCC, we also find limited overlap between GO terms for significantly affected genes across the data layers (Fig. 6b).

Next, we performed SiRCle clustering on the PanCan data comparing tumour versus normal, thus integrating the DNA-methylation, mRNA and protein layers to recover shared functions across the multi-omic layers. First, assessing the number of genes falling into the different SiRCle clusters, we found distinct genes and hence biology in the PanCan cohort as compared with the ccRCC cohort (light grey, Fig. 6c). However, there were also some overlaps with the ccRCC cohort (dark grey, Fig. 6c). Hypomethylation (SiRCle cluster MDE) affects genes important in extracellular matrix organisation in both ccRCC and PanCan (Fig. 6d), whilst it additionally drives immune response in ccRCC (Fig. 6e). Interestingly, the SiRCle cluster MDS_TMDE includes tissue-specific terms for both ccRCC and PanCan that are related, in the case of ccRCC, to renal function and in case of PanCan for lung, heart and digestive system (Fig. 6f). Thus, SiRCle enables to understand on which layer tissue specific characteristics are regulated across cancers.

We next tested for multi-omic changes between late and early-stage patients and find genes implicated in patient survival across the PanCan cohort. We found minimal significant changes between the early and late-stage patients in DNA methylation, RNA and protein expression. Besides O-glycan biosynthesis, no major metabolic pathways were significantly different between late and early-stage patients across their multi-omic profile in the PanCan cohort (Fig. 6g). This result further underlines the peculiar metabolic rewiring of renal cancer, in which we found multiple metabolic pathways being affected, and hence it suggests that the multi-omic metabolic signatures we identified in ccRCC, such as methionine metabolism, are indeed specific to ccRCC (Fig. 5a–b, d). Next, we tested the genes from the O-glycan biosynthesis pathway for its impact on survival using GEPIA2, a tool for large-scale expression profiling and analysis [66], across all TCGA cancers. We found that GALNT7, an enzyme that controls the initiation step of protein glycosylation and transfer of N-acetylgalactosamine to serine and threonine amino acid residues [67], is repressed in late-stage tumours and is implicated in patient survival across cancers (Fig. 6h). Conversely the genes from O-glycan biosynthesis in the SiRCle cluster MDE_ TMDS, namely *GALNT14*, *GCNT3* and *B3GNT6*, improve patients’ survival when repressed (Fig. 6h). Our findings show that both the PanCan and ccRCC comparative analyses enable genes to be identified that are implicated in patient survival in addition to extracting the gene’s level of regulation, highlighting a new way to investigate genes changing in a multi-omic context.

Since the development of SiRCle, an independent ccRCC patient’s cohort dissecting the intra-tumour heterogeneity (ITH) was released [47]. As such, we investigated how robust our findings are in comparison to this heterogeneous ccRCC cohort performing similar pre-processing methods and the same SiRCle parameters (see “Methods” for details). We find a higher conservation of both genes and ORA functions as compared with the PanCan cohort (Fig. 6, Table S11). Of the metabolic genes such as *ALDOA*, *PKM* or *ENO1/2* in Fig. 3, we find 70% of these are assigned to the same cluster in the new ccRCC cohort, whilst only 12% of these are assigned the same cluster in the PanCan cohort. Our results highlight the reproducibility of the SiRCle analysis when considering the same cancer.

## Discussion

Here, we present SiRCle, a method for integrating DNA methylation, RNA-seq and proteomics data to extract support on which layer or layers (DNA methylation, transcription and/or translation) genes are regulated and/or perturbed between patient subsets with different phenotypic traits (Fig. 6). We applied SiRCle to a case study of ccRCC and we demonstrate that it extends to other cancers, using a PanCan cohort. ccRCC is a particularly heterogeneous cancer with cascades of mutations and complex remodelling of the tumour microenvironment that affect multiple regulatory networks [3], hence we expect many regulatory relationships to hinge on joint consideration of multiple data modalities, some being specific to ccRCC and others being in line with the PanCan cohort.

Across a cohort of ccRCC patients, we show modest correlation between data of different modalities as well as limited agreement among biological terms extracted from each layer in isolation. This observation underscores the challenges of analyses applied to individual omics since some of the gene changes observed at the epigenetic or transcriptional layer may not be translated into protein changes. SiRCle was developed to group genes using regulatory “flows” based on differences between control and disease at each layer, and we found the majority of genes exhibit states counter to the generalisation that increased DNA methylation at the gene represses transcription, which in turn decreases translation of the gene product within the condition, or (conversely) that reduced methylation at the gene activates transcription, which in turn increases translation of its product. Whilst the extent of dissonance highlights the importance of considering multiple layers of regulation, we found that by grouping via cross-layer flows elicited distinct biological terms, e.g. metabolism and immune response, subsuming the function extracted from each layer in isolation. The advantage of SiRCle clustering is that by considering all possibilities of regulation, we can pinpoint the likely layer at which genes are dysregulated in disease.

In addition to consolidating biological function across the layers, SiRCle clustering captured relationships from adjunct data, such as TF relationships and metabolic changes. We found TF’s targets fell predominantly in clusters regulated on the layer of transcription or methylation and were able to distinguish HIF TF targets that increased via transcription, from those that increased via hypomethylation. The advantage of performing the regulatory clustering prior to the TF analysis is that we can exclude TFs that are predicted to drive genes in clusters regulated at the layer of translation (e.g. TMDE, TMDS), given TFs work at the layer of transcription. Coupling SiRCle annotations with metabolic activity, we were able to establish a more detailed view of how changes in regulation correspond to changes in metabolic pathways. We found that glycolysis was regulated on the methylation layer (MDE), whilst the TCA cycle and oxidative phosphorylation were regulated translationally (TMDS). We noticed that glycolytic enzymes are upregulated in the context of ccRCC, suggesting hypomethylation increases DNA accessibility [63], potentially improving the capacity for HIF1A to bind and upregulate glycolytic enzyme transcription and eventual translation. However, these findings require validation by more detailed DNA methylation analysis, such as whole genome bisulphite sequencing, given our work infers the DNA methylation state based on probe assays, which alone do not capture the full methylation profile. Hakimi et al. showed that metabolites of the glycolysis pathway split at the level of GAPDH, whereby metabolites upstream of this enzyme are accumulated, and the ones downstream are depleted [7]. We found that this is not in line with the enzyme expression, yet this counterintuitive finding could be explained by the fact that the catalytic function of many enzymes is altered by post-translational modifications such as phosphorylation (e.g. PGK1 [68] and PKM [69]) or oxidation (e.g. GAPDH [70]). In fact, it has been shown for the CPTAC patient cohort that *PGK1* and *PKM* have increased phosphorylation when comparing tumour versus normal [14]. Moreover, glycolytic flux towards lactate in ccRCC is inhibited by *FBP1* [71], yet *FBP1* is not mutated in this patient cohort.

Until this point, we investigated changes across the whole ccRCC cohort, however, there are distinct subpopulations of patients, with differing prognoses, for example those with early or late-stage tumours. We sought to investigate the regulatory differences between patient subpopulations, however, when we analysed each layer independently there was limited overlap between functional terms. Thus, to find regulatory differences between patient subpopulations (e.g. stage), we developed a novel integration approach using a VAE to learn a single key feature from the DNA methylation, mRNA and protein layers for each SiRCle cluster. By integrating across the layers prior to performing statistical analyses, we found significant differences in genes important for survival when comparing early and late-stage patients and showed that the VAE produces biologically meaningful integrated values. Thus, the VAE in combination with SiRCle can also be applied to other patient subpopulations such as age, gender or mutation pattern. Moreover, we showed that our integrative approach can be used to perform GSEA between patients’ groups. SiRCle allowed us to identify key differences between patients’ subpopulations in terms of metabolic rewiring, such as oxidative phosphorylation being less suppressed in late-stage patients. We also observed that within the TCA cycle intermediates, citrate and aconitate were depleted in late-stage tumours, despite the increased expression of the enzymes involved in their biosynthesis. Whether this result indicates a further functional regulation of the TCA at the post-translational layer, as for instance PDH phosphorylation, which in turn controls citrate biosynthesis, needs to be further investigated. In addition, we observed an increase in succinate in late-stage patients and this finding could indicate that these tumours may be more hypoxic, considering the role of succinate as marker of low oxygen in tissue [72]. The increase in succinate could also be due to the need to generate NADH, where the TCA cycle fuels into succinate and the heme biosynthesis pathway as previously described [73]. In line with this, HMOX1 is upregulated, likely via hypomethylation, and the metabolites heme and bilirubin are also accumulated (Fig. 3a). As discussed by Hakimi et al., the TCA cycle could be fuelled via glutamate, which fuels into citrate via reductive carboxylation [7]. Lastly, in line with the enzyme expression, serine levels are depleted (Fig. 3a). Whilst similarities in metabolic profiles between the early and late-stage patients were identified, we also found differences in methionine metabolism.

SiRCle is broadly applicable to other datasets and contexts. Indeed, when we apply SiRCle to a PanCan dataset we find that across both the PanCan and ccRCC datasets extracellular matrix genes are regulated by hypomethylation (SiRCle cluster MDE). We also observed that tissue specific genes are regulated via hypomethylation and/or enhanced translation (SiRCle cluster MDS_TMDE) for both cohorts suggesting that SiRCle can elucidate the data layer responsible for the regulation of tissue identity. Additionally, distinct biological processes are identified, such as the ccRCC immune response being driven by hypomethylation. When we consider cascading events leading to the transition from early to late-stage tumours, we find that the metabolic rewiring in ccRCC appears distinct, or at least cancer specific. Whilst we focused on the impact of DNA methylation and the corresponding downstream effects, the SiRCle logic and framework could be extended and applied to other events that drive cancer such as copy number variation (CNV) and histone modifications. For example, the logical rules implemented expect an inverse relationship between epigenetics and the subsequent layers which could directly apply to repressive marks such as H3K27me3. To extend this further, in future work a positive relationship could be added instead as would be necessary for positive marks such as H3K27ac [74]. In this context, it is also important to mention that ccRCC is associated with several, frequent CNVs. As SiRCle does not take into account CNVs, it would be beneficial to further dissect the involvement of CNV in a gene’s expression by developing dedicated methods in the future.

With SiRCle, we opted for a simple and logical approach with no bias on directionality of effect. However, the result of analyses does depend on upstream choices and therefore outcomes should be interpreted with caution. The choice of methodology to assign DNA methylation changes to genes will affect the outcome, as in most post hoc multi-omic analyses. The specifics of upstream analyses, such as the choice of differential analysis tool, could also have an impact on the results as such it is prudent for the user to consider their system under study when choosing differential expression analysis and differential methylation analysis (e.g. EdgeR, DESeq, limma) approaches preceding SiRCle. Similarly, as the choice of filtering of genes and samples are specific to the biological question and technical setup, the filtering in SiRCle is up to the user and we merely show one example of pre-processing specific for the data used in this study. We also note that the choice of cutoff in SiRCle, for DNA methylation, protein and gene expression, will affect the sensitivity of identifying regulatory changes and will be dataset dependent. Whilst we find that across two ccRCC cohorts, using the same cutoffs works effectively, we suggest users consider the nuances of their system of study, the distribution of the changes across their dataset and the sensitivity of the assay being performed.

A specific technical limitation of public patient data is missing information or assays. Using a VAE, missing samples can be retained by using imputed values, and whilst in our analyses we opted to fill with the mean value, other more complex imputation techniques could increase the statistical strength of the integrated value. These choices are ultimately up to the user, however, our pipeline is what we deemed to be “general and all purpose”, and as we shown, functions in both a specific cancer and in a PanCan context. Finally, as with any statistical test, the significance of the integrated test is dependent on the number of samples in each comparison group, which means that important changes may be lost when we are comparing groups with small sample sizes. A major limitation is also the lack of other epigenetic data such as copy number variation (CNV), histone modifications and other epigenetic information limiting the biological conclusions that can be drawn from the effects of DNA methylation on the gene expression. Furthermore, we note that the biological findings from our PanCan analyses are dependent on an incomplete cohort of cancer patients. Whilst we found that our ccRCC cohort SiRCle clusters showed conservation to another more heterogeneous ccRCC cohort, we believe a follow-up study to extend the PanCan findings by inclusion of further cancer types could extend our findings further.

## Conclusions

Our results highlight SiRCle’s ability to reveal potential mechanisms of phenotype regulation in cancer, both specifically in ccRCC and broadly in a PanCan context. Using SiRCle, researchers can extend known relationships, find regulators and drivers of gene clusters and extract genes that differ between patient cohorts in many different cancer types. We propose that SiRCle can be applied to other diseases and ranks genes according to biological features.

## Supplementary Information

Additional file 1: Supplementary figures (Figs. S1–S4).

Additional file 2: Table S1 Clinical information of patients from each study. Sheet 1: DNA methylation clinical data from TCGA for ccRCC. Sheet 2: Protein clinical and aliquot data from CPTAC for ccRCC. Sheet 3: RNA clinical data from TCGA for ccRCC. Sheet 4: DNA methylation clinical data from TCGA for PanCan. Sheet 5: Protein clinical and aliquot data from CPTAC for PanCan. Sheet 6: RNA clinical data from TCGA for PanCan.

Additional file 3: Table S2 DE results for each dataset and results from the SiRCle clusters. Sheet 1: SiRCle results ccRCC. Sheet 2: ORA TvN for DNA methylation, RNA and protein datasets. Sheet 3: ORA SiRCle clusters. Sheet 4: Protein DA. Sheet 5: RNA DE. Sheet 6: CpG DMC. Sheet 7: Protein TvN metabolic pathways. Sheet 8: RNA TvN metabolic pathways. Sheet 9: CpG TvN metabolic pathways.

Additional file 4: Table S3 TF analysis output. Sheet 1: Transcription factor output table.

Additional file 5: Table S4 Metabolomics data for tumour vs normal. Sheet 1: Clinical information for metabolomics patients. Sheet 2: TvN differential expression results. Sheet 3: GSEA on protein TvN for metabolic pathways. Sheet 4: Comparison of stage IV vs stage I. Sheet 5: Comparison of young vs old.

Additional file 6: Table S5 ORA and DE, DA and DMC for early and late analyses. Sheets annotated with labels.

Additional file 7: Table S6 Integrated dataset for patients. Sheet 1: Patient information for ccRCC patients. Sheet 2: Comparison of late and early patients. Sheet 3: Late and early KEGG pathways. Sheet 4: Late and early metabolic pathways. Sheet 5: Late and early statistics.

Additional file 8: Table S7. Sheet 1: Benchmarking results.

Additional file 9: Table S8 SiRCle ORA PanCan. Sheet 1: SiRCle results PanCan. Sheet 2: ORA for tumour vs normal for CpG, protein and RNA. Sheet 3: ORA for each SiRCle cluster. Sheet 4: Protein DA results. Sheet 5: RNA DE results. Sheet 6: CpG differential methylation results.

Additional file 10: Table S9 ORA for early and late analysis PanCan. Sheets annotated with labels.

Additional file 11: Table S10 Integrated dataset for PanCan analysis. Sheet 1: Patient information for PanCan patients. Sheet 2: Comparison of late and early patients. Sheet 3: Late and early KEGG pathways. Sheet 4: Late and early metabolic pathways. Sheet 5: Late and early statistics.

Additional file 12: Table S11 New ccRCC cohort with DE results, SiRCle and ORA. Sheet 1: DE methylation. Sheet 2: DE RNA. Sheet 3: DE protein. Sheet 4: SiRCle. Sheet 5: ORA on each SiRCle cluster.

## Data Availability

No original data were collected as part of this study; the studies where data were collected from and that are included in this paper are as follows: Clark et al. (PDC000127) [14], Hakimi et al. [7], ccRCC-ITH [47], Head and Neck Squamous Cell Carcinoma (PDC000221 [48]), Lung Adenocarcinoma (PDC000153 [49]), Lung Squamous Cell Carcinoma (PDC000234 [50]) and Pancreatic Ductal Adenocarcinoma from CPTAC (PDC000270 [51]). Collection for CPTAC studies was done via the online portal and collection of the raw data can be reviewed in each of these manuscripts (see “Methods”). The processed datasets (PanCan) and ccRCC are available at Zenodo (Mora and Schmidt, PanCan data https://zenodo.org/records/8284067, 2024) [75]. Data generated as part of this study are available as supplementary tables available on the GitHub, https://github.com/ArianeMora/SiRCle_multiomics_integration, [76] where information to download all raw data from the respective studies is available. If there is any additional information required for reanalysis of the data reported in this paper contact christina.schmidt1@outlook.de and ariane.n.mora@gmail.com. The SiRCle method described in this manuscript is available as a Python package on GitHub (https://github.com/ArianeMora/scircm) [77], along with source code installation instructions and documentation are available (https://github.com/ArianeMora/SiRCle_multiomics_integration) [76]. If there is any additional information required for reanalysis or code adaptations, please post an issue on the GitHub.

## Abbreviations

ccRCC: Clear cell renal cell carcinoma
TF: Transcription factor
VHL: Von Hippel-Lindau
mTOR: Mammalian target of rapamycin
CPTAC: Clinical Proteomic Tumor Analysis Consortium
VAE: Variational autoencoder
RNA: Ribonucleic acid
ATAC: Assay for transposase-accessible chromatin
DNA: Deoxyribonucleic acid
mRNA: Messenger RNA
GWAS: Genome-wide association studies
PCA: Principal component analysis
SiRCle: Signature Regulatory Clustering
TCGA: The Cancer Genome Atlas
ITH: Intra-tumour heterogeneity
BG: Background method
RG: Regulation Grouping
w.r.t.: With respect to
log_2_*FC*: Log base twofold change
BH FDR: Benjamini-Hochberg false discovery rate
ORA: Over representation analysis
PDC: Proteomic Data Commons
GSEA: Gene set enrichment analysis
GO: Gene Ontology
ChIP-seq: Chromatin immunoprecipitation sequencing
ETC: Electron transport chain
GSH: Cysteine methionine and glutathione
PanCan: Pan Cancer
MDS: Methylation Driven Suppression
TMDS: Translation and translational-modification-Driven Suppression
TMDE: Translation and translational-modification-Driven Enhancement
MDE: Methylation Driven Enhancement
TPDE: Transcriptional and Processing-Driven Enhancement
TPDS: Transcriptional and Processing-Driven Suppression
