# Supplementary material for: SiRCle (Signature Regulatory Clustering) model integration reveals mechanisms of phenotype regulation in renal cancer
Source: Genome Med. 2024 Dec 4;16:144. doi: 10.1186/s13073-024-01415-3 (PMC11616309; doi:10.1186/s13073-024-01415-3)
Supplement: Supplementary file 1 — Additional file 1: Supplementary figures (Figs. S1–S4). [file 13073_2024_1415_MOESM1_ESM.pdf]

Additional File 1

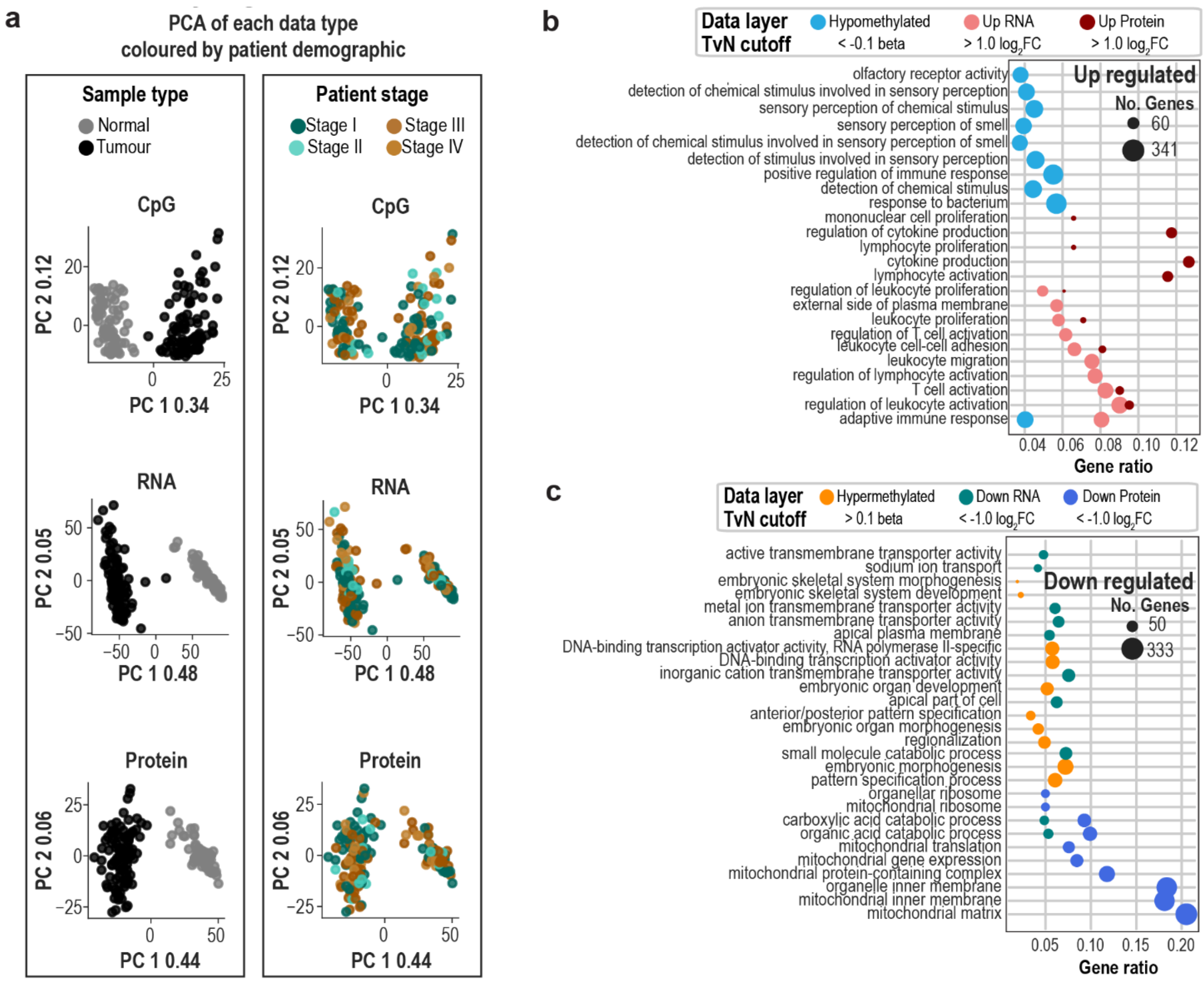

Fig. S1: Demographics and SiRCle clusters.

a, Case annotations based on sample and tumour stage visualised on PCA. **b-c,** The top 10 gene ontology terms from over representation analysis for each data layer are visualised. Where two dots appear on the same line, it means they were both top terms for that data layer. This only occurs for the up regulated terms, i.e. the terms that were associated with an increase on the protein or mRNA layers. Data layer refers to the data layer on which the differential analysis was run, and TvN cut-off denotes the cut-off used when selecting genes for ORA. **c**, as in **b**, except with the bottom terms.

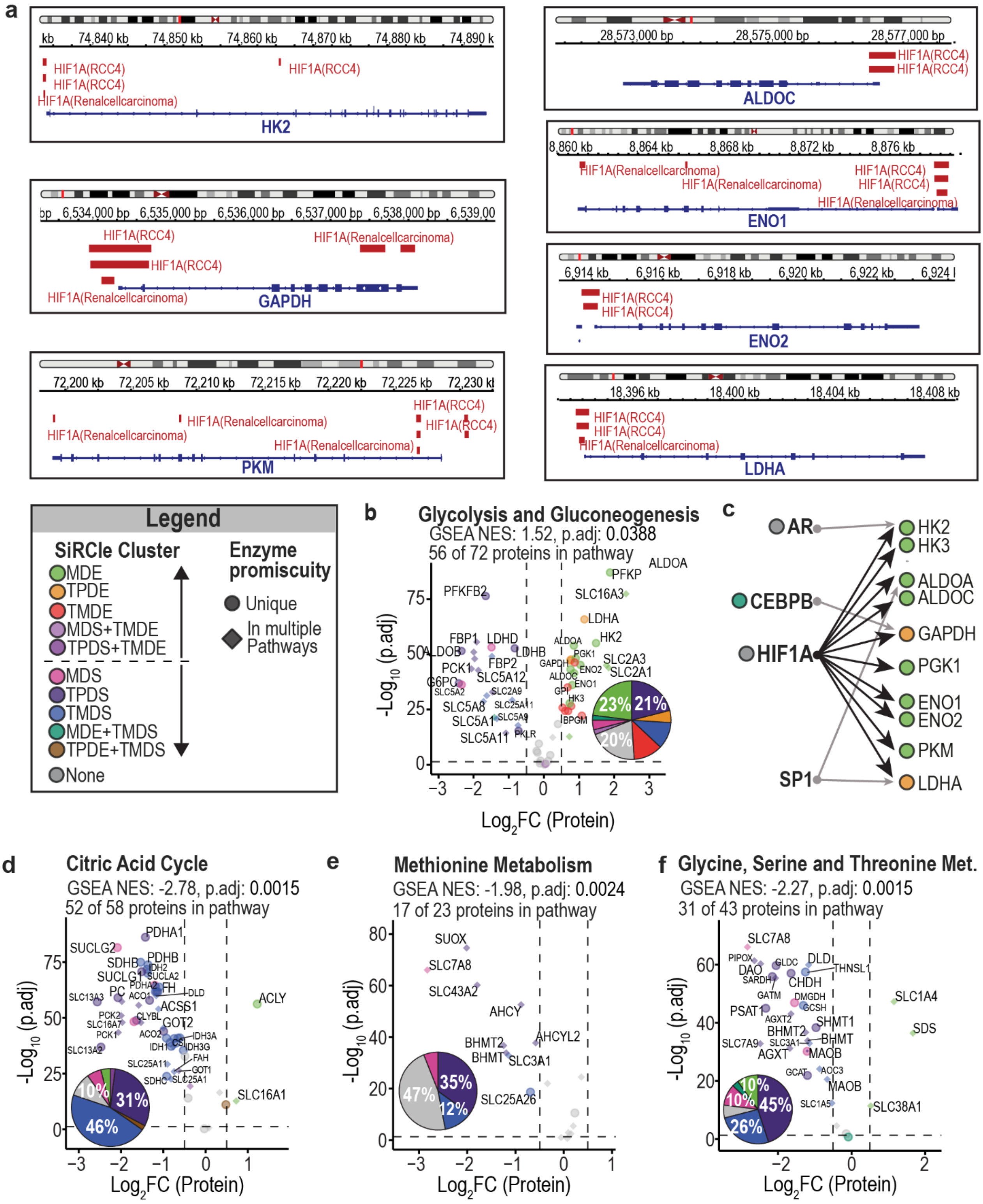

Fig. S2: TF drivers.

a, HIF1A ChIP-seq peaks binding sites at glycolytic enzyme TSS's. **b,** Volcano plots are based on the metabolic signatures and the number of proteins detected of the pathway are reported. Proteins that are unique for the metabolic pathways are displayed in circles and proteins that are part of multiple metabolic pathways are displayed in diamond. The colour code depends on the SiRCle cluster the protein is part of and is summarised in the pie chart. The protein log2FC is calculated between tumour versus normal. **b,** Volcano plot of glycolysis and gluconeogenesis. **c,** Transcription factor (TF) factor network of TFs from manually curated repositories that drive glycolytic enzymes in the cluster Methylation Driven Enhancement, MDE and Transcription and Processing Driven Enhancement, TPDE., **d,** Volcano plot of the citric acid cycle. e, Volcano plot of methionine metabolism. **f,** Volcano plot of glycine, serine and threonine metabolism.

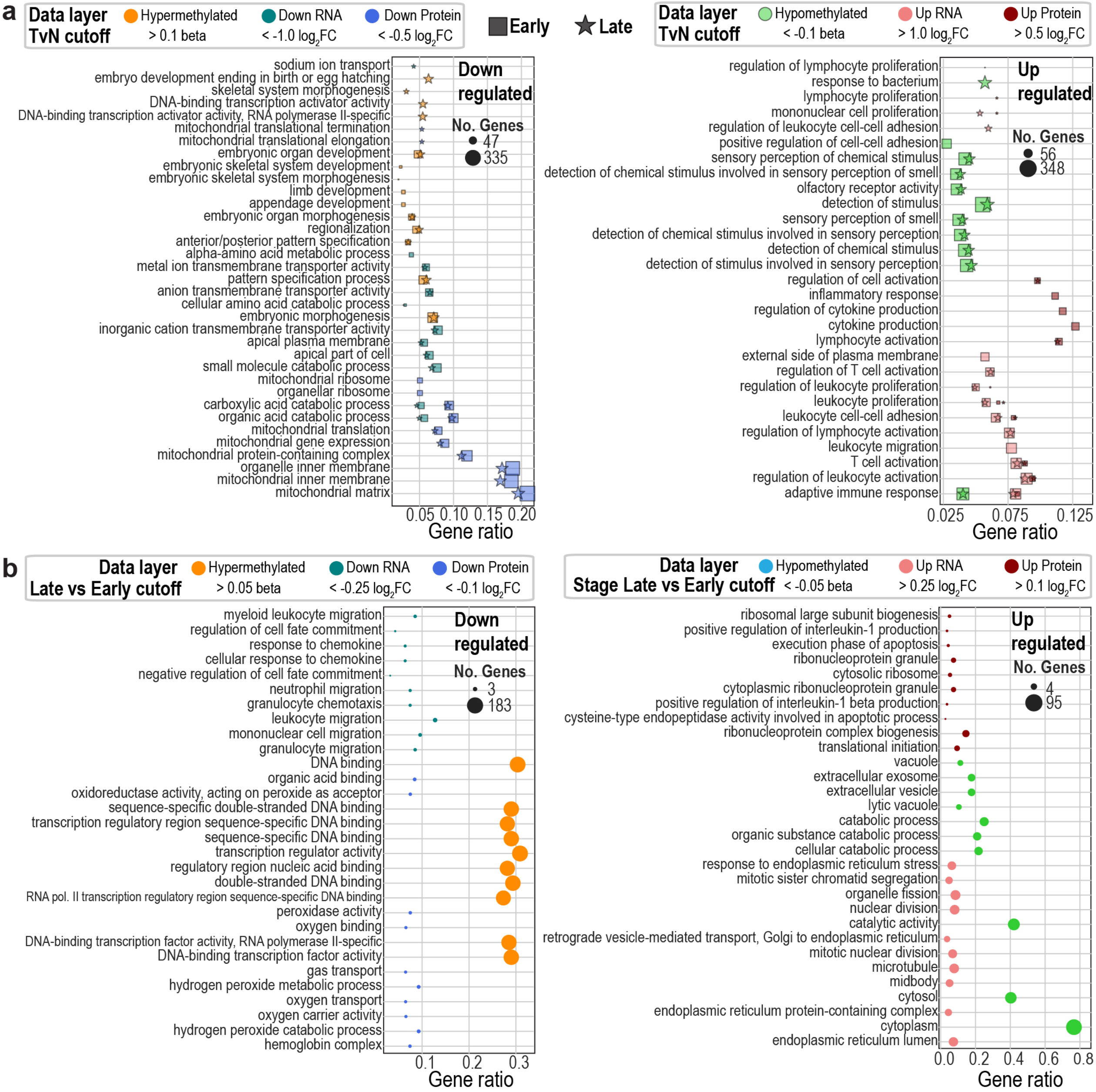

Fig. S3: Late vs Early results.

a, Top 10 gene ontology (GO) terms from over representation analysis (ORA) for each data layer are visualised for early tumour vs normal and late tumour versus normal (TvN) on the same plot. Where two dots appear on the same line, it means they were both top terms for that data layer or in both early and late TvN comparisons. Data layer refers to the data layer on which the differential analysis was run and the state, for example increased or decreased in tumour (Up and Down respectively), and TvN cut-off denotes the TvN cut-off used when selecting genes for ORA. **b,** Top 10 GO terms from ORA for each data layer are visualised for late tumour vs early tumours.

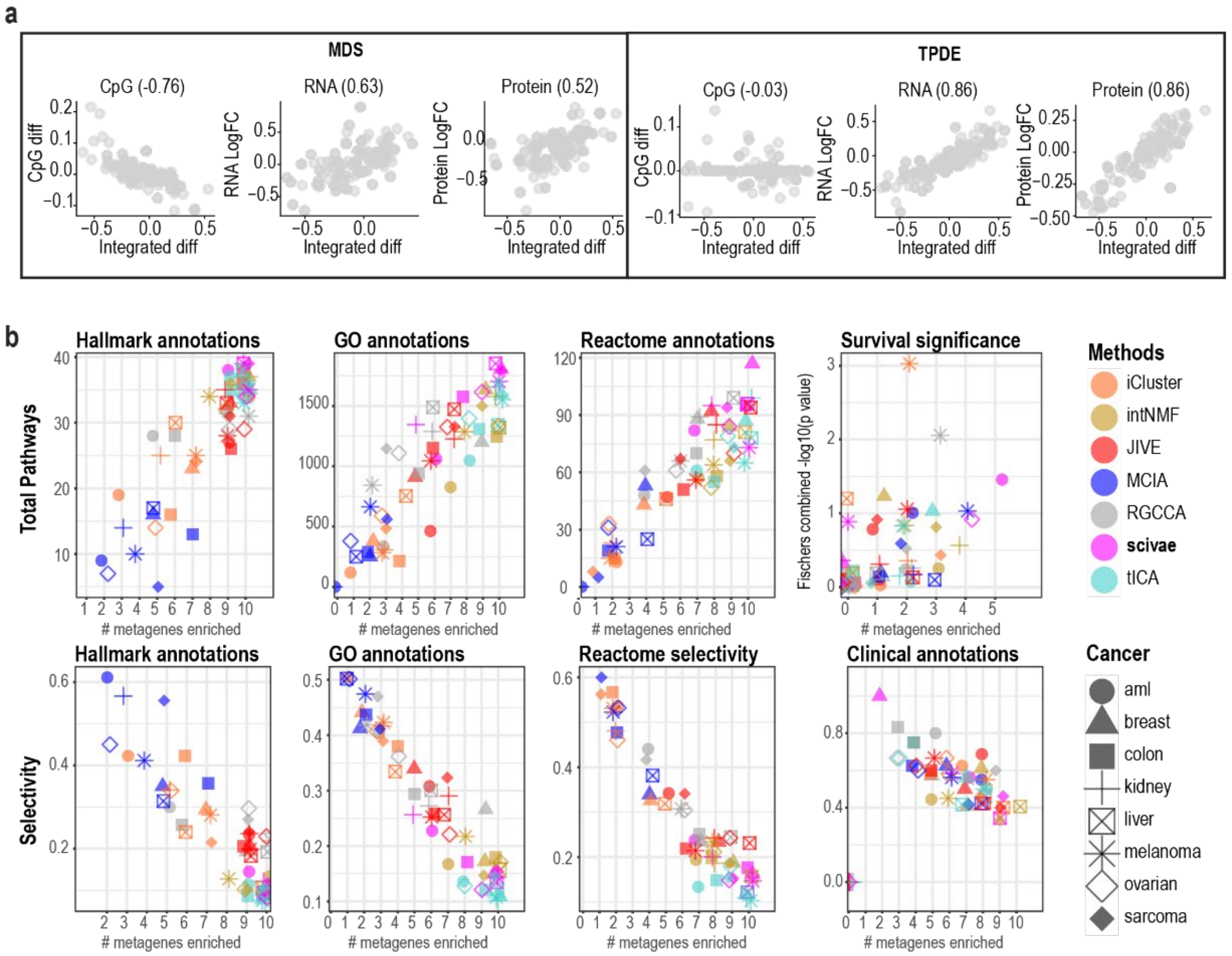

Fig. S4: VAE integrated value.

a, Spearman's correlation between the "real difference" in each data type and the integrated difference for two clusters, MDS and TPDE for the ccRCC cohort. **b,** Benchmarking was performed using momix, with each tool coloured by method, and each cancer by shape. GSEA analysis was performed on each dimension, where Metagene corresponds to a latent dimension. Benchmarking against the tools we see that scivae consistently records more pathways than the other tools for many of the cancers, yet has a lower performance on clinical annotations. Despite having a high total number of pathways, scivae also reports a high selectivity with only 2 latent features.
